# Deep Learning for Ultrasound Image Formation: CUBDL Evaluation Framework and Open Datasets

**DOI:** 10.1109/TUFFC.2021.3094849

**Published:** 2021-11-23

**Authors:** Dongwoon Hyun, Alycen Wiacek, Sobhan Goudarzi, Sven Rothlübbers, Amir Asif, Klaus Eickel, Yonina C. Eldar, Jiaqi Huang, Massimo Mischi, Hassan Rivaz, David Sinden, Ruud J. G. van Sloun, Hannah Strohm, Muyinatu A. Lediju Bell

**Affiliations:** Department of Radiology, Stanford University, Stanford, Ca 94305 USA; Department of Electrical and Computer Engineering, Johns Hopkins University, Baltimore, MD 21218 USA; Department of Electrical and Computer Engineering, Concordia University, Montreal, QC H3G 1M8, Canada; Fraunhofer Institute for Digital Medicine MEVIS, 28359 Bremen, Germany; Department of Electrical Engineering and Computer Science, York University, Toronto, ON M3J 1P3, Canada; Department of Physics and Electrical Engineering, University of Bremen, 28359 Bremen, Germany; Department of Mathematics and Computer Science, Weizmann Institute of Science, Rehovot 7610001, Israel; Department of Biomedical Engineering, Johns Hopkins University, Baltimore, MD 21218 USA; Department of Electrical Engineering, Eindhoven University of Technology, 5612 Eindhoven, The Netherlands; Department of Electrical and Computer Engineering, Concordia University, Montreal, QC H3G 1M8, Canada; Fraunhofer Institute for Digital Medicine MEVIS, 28359 Bremen, Germany; Department of Electrical Engineering, Eindhoven University of Technology, 5612 Eindhoven, The Netherlands, and also with Philips Research, 5656 Eindhoven, The Netherlands; Fraunhofer Institute for Digital Medicine MEVIS, 28359 Bremen, Germany; Department of Electrical and Computer Engineering, the Department of Biomedical Engineering, and the Department of Computer Science, Johns Hopkins University, Baltimore, MD 21218 USA

**Keywords:** Beamforming, channel data, deep learning benchmark, evaluation metrics, neural networks, open science, sound speed estimation, ultrasound image formation

## Abstract

Deep learning for ultrasound image formation is rapidly garnering research support and attention, quickly rising as the latest frontier in ultrasound image formation, with much promise to balance both image quality and display speed. Despite this promise, one challenge with identifying optimal solutions is the absence of unified evaluation methods and datasets that are not specific to a single research group. This article introduces the largest known international database of ultrasound channel data and describes the associated evaluation methods that were initially developed for the challenge on ultrasound beamforming with deep learning (CUBDL), which was offered as a component of the 2020 IEEE International Ultrasonics Symposium. We summarize the challenge results and present qualitative and quantitative assessments using both the initially closed CUBDL evaluation test dataset (which was crowd-sourced from multiple groups around the world) and additional *in vivo* breast ultrasound data contributed after the challenge was completed. As an example quantitative assessment, single plane wave images from the CUBDL Task 1 dataset produced a mean generalized contrast-to-noise ratio (gCNR) of 0.67 and a mean lateral resolution of 0.42 mm when formed with delay-and-sum beamforming, compared with a mean gCNR as high as 0.81 and a mean lateral resolution as low as 0.32 mm when formed with networks submitted by the challenge winners. We also describe contributed CUBDL data that may be used for training of future networks. The compiled database includes a total of 576 image acquisition sequences. We additionally introduce a neural-network-based global sound speed estimator implementation that was necessary to fairly evaluate the results obtained with this international database. The integration of CUBDL evaluation methods, evaluation code, network weights from the challenge winners, and all datasets described herein are publicly available (visit https://cubdl.jhu.edu for details).

## I. Introduction

SIGNIFICANT research has been dedicated recently to developing methods for deep learning in ultrasound imaging, as summarized in several recent review articles and special issue editorials [1]–[4]. The merger of deep learning and ultrasound image formation is promising because it has the potential to shed light on features that are not considered by algorithmic approaches that underlie the mathematical, model-based component of image formation, with multiple input-output and training options [5]–[7]. These data-driven deep learning approaches have the potential to be more robust than the traditional model-based beamforming methods, as they do not require parameter changes when switching to different scanners, they are able to generalize across different datasets, and they can infer from advanced beamforming methods in less time than that required to perform the otherwise computationally intensive calculations associated with advanced beamformers [8]–[11]. Despite the promising potential of deep learning approaches applied to ultrasound imaging, there has been a noticeable dearth of publicly available frameworks to evaluate new deep learning methods with the same reference data. Such open frameworks are useful for benchmarking and comparing methods against each other, as demonstrated in the fields of visual recognition [12] and computed tomography [13].

One outcome of the Challenge on Ultrasound Beamforming with Deep Learning (CUBDL), offered as a component of the 2020 IEEE International Ultrasonics Symposium, was the development of an open evaluation framework that may be used as a standard for deep learning image formation methods in ultrasound imaging. The overarching goal of this challenge was to explore the potential of deep learning to improve ultrasound image quality and to balance these improvements with implementation practicality (i.e., reasonable display frame rates) [14]. Comparisons were completed on the same datasets and with the same evaluation methods and framework.

This article summarizes and compares submissions to CUBDL using the evaluation framework developed for this challenge. We present results on both the initially closed CUBDL evaluation test dataset and additional *in vivo* breast ultrasound data contributed after the completion of the challenge. Components of this evaluation framework were publicly available while CUBDL was in progress, and the framework is now merged with the CUBDL test data and the additional datasets described herein. This framework merger and the associated datasets are publicly available at [15] and [16], respectively (visit https://cubdl.jhu.edu for more details). The summary within this article and the accessibility of our data and code are provided in efforts to enable future benchmarking and evaluation of new methods.

The remainder of this article is organized as follows. Section II provides an overview of the CUBDL challenge, including dates associated with major milestones. Section III describes details of the open datasets, the top challenge submissions, the CUBDL evaluation process, scoring and ranking methods, and the post-CUBDL analyses implemented to ensure robustness and reproducibility of our shared code and challenge winner declaration. This section is organized to align with the sequential timeline of events and is effectively a combination of materials used and methods implemented by three categories of contributors to this article: 1) the CUBDL organizers who implemented dataset curation, evaluation, scoring, assessment, and rankings; 2) the top challenge participants who provided network details; and 3) A.W. who completed the independent post-CUBDL evaluations and curation of the post-CUBDL datasets. Section IV presents the results from the perspective of the CUBDL organizers, detailing our evaluation and analysis findings, with post-CUBDL contributions from A.W. Section V discusses results within the broader context of the current state of the field from the perspective of the CUBDL organizers. Finally, Section VI concludes the article.

## II. Challenge Summary and Timeline

Organization of this challenge initiated on October 13, 2019. The complete challenge setup, submission process, and discussion of the challenge organization are described in detail in the associated conference proceedings coauthored by the challenge organizers [20]. To briefly summarize details in [20] that are relevant to this article, the challenge was designed with no specific training data provided by the CUBDL organizers, because a review of current literature on the topic of deep learning for ultrasound beamforming reveals that there are multiple possibilities for both training approaches and training datasets. Therefore, the CUBDL organizers decided to keep the training open-ended, with the goals of encouraging a wide variety of submission approaches, enabling participants to innovate on the training methods, and challenging participants to produce a network that achieved specific tasks and met specified requirements. The publicly available PICMUS data [21] were additionally advertised and considered to serve as possible training data for participants who desired more structure. From November 22, 2019 to January 23, 2020, test data were crowd-sourced from multiple groups around the world, representing the largest known international database of ultrasound channel data.

The challenge opened to participants on January 30, 2020, and closed to participants on the submission deadline of June 23, 2020. Although three optional tasks were conceived by the CUBDL organizers [20] and advertised on the CUBDL website [14], all participants submitted networks to be evaluated for Task 1, which was beamforming with deep learning after a single plane wave transmission, with two optional subtasks. Task 1a was explicitly focused on creating a high-quality image from a single plane wave to match a higher quality image created from multiple plane waves. Three participants submitted to this subtask. Task 1b allowed more flexibility to create images with the highest possible image quality metrics, including metrics that exceeded those obtained with multiple plane wave transmissions (e.g., no speckle preservation required). One participant submitted to this subtask. Note that the dates that CUBDL was open to participants overlapped with the onset and peaks of the global COVID-19 pandemic [22], which likely affected participation rates.

Participants were instructed to train networks using their preferred machine learning frameworks (e.g., TensorFlow, PyTorch) and submit final model files to the IEEE DataPort website [16]. The CUBDL organizers then downloaded the submitted models and launched a Python script to perform evaluation [15] on the internationally crowd-sourced database of test data [16]. Evaluation metrics advertised since the launch of the CUBDL website [14] were pre-selected by the CUBDL organizers based on literature from multiple groups reporting beamforming with deep learning (e.g., [8], [9], [11], [23]) and based on common computer vision literature containing assessments of network complexity (e.g., [24]–[26]).

After evaluation, two participants (one submitting to Task 1a and the other submitting to Task 1b) produced networks that showed evidence of overfitting to the training data when tested on the closed CUBDL test dataset for Task 1 [19], [27]. These participants were declared the runners up of the challenge. Details about their networks and performance results are available in [19] and [27] and in the presentation document published on the CUBDL website [14], which was initially prepared for the live CUBDL challenge session on September 11, 2020, at the 2020 IEEE International Ultrasonics Symposium. The remaining two network submissions [28], [29] are compared and evaluated in greater detail in this publication, which upholds the commitment advertised on the CUBDL website that only top submissions would be included in the journal publication associated with the challenge [14]. Also as promised on the CUBDL website, the trained model weights for these top submissions are available with the public release of the CUBDL evaluation code [15] and datasets [16].

## III. Materials and Methods

### A. Open Datasets

#### 1) CUBDL Task 1 Dataset:

The test data for Task 1 of the challenge consisted of 21 image acquisition sequences crowd-sourced from six institutions, referenced hereafter by the short-hand three-letter code provided in parentheses: 1) Department of Biomedical Engineering, Tsinghua University, Beijing, China (TSH); 2) Department of Radiology, Mayo Clinic, Rochester, MN, USA (MYO); 3) Microelectronic Systems Design Laboratory, University of Florence, Florence, Italy (UFL); 4) Signal Processing Systems group, Eindhoven University of Technology, Eindhoven, The Netherlands (EUT); 5) CREATIS, INSA Lyon, Lyon, France (INS); and 6) Research Group for Digital Signal Processing and Image Analysis, University of Oslo, Oslo, Norway (OSL).

The test data from these institutions consisted of 21 image acquisition sequences from targets including phantoms, simulation, and an *in vivo* brachioradialis. The phantom data consisted of a total of 9 different phantoms from three manufacturers including: 1) CIRS models 040, 049, 054GS, 050, and ATS549; 2) GAMMEX models 404GSLE, 403, and 410 SCG; and 3) NPL Thermal Test Phantom. Two MYO phantom acquisitions included a layer of *ex vivo* porcine abdominal tissue to introduce acoustic clutter. The *in vivo* data were acquired after informed consent and ethics approval. This wide range of channel data was acquired with three ultrasound scanner models (i.e., Verasonics Vantage 128, Verasonics Vantage 256, and ULA-OP 256) and six ultrasound transducer models. The acquisition center frequencies ranged from 3.1 to 8 MHz. The sampling frequencies ranged from 6.25 to 78.125 MHz. The ultrasound transducers consisted of linear and phased arrays.

Each contributed dataset for Task 1 consisted of acquisitions from 31 or 75 steered plane waves, with transmission angles ranging −15° to 15° or −16° to 16°. Additional summary details and acquisition parameters are reported in Table I, along with references to reports in which the data first appeared. The full list of sequence numbers and more specific details are available in the Appendix, which mirrors a majority of the information that was provided in the CUBDL Data Guide while the challenge was open.

#### 2) JHU In Vivo Breast Dataset for Post-CUBDL Evaluation:

To improve the variety of *in vivo* datasets, the Photoacoustic and Ultrasonic Systems Engineering Laboratory, Johns Hopkins University, Baltimore, MD, USA (three-letter code: JHU), contributed additional test data for network evaluation after the challenge was closed. These *in vivo* breast ultrasound data (initially reported by Li *et al.* [19]) consisted of 11 acquisition sequences, acquired from six patients, including two orthogonal scans (i.e., radial and anti-radial) of five patients and a radial scan of one patient. These data were acquired after informed consent and with approval from the Johns Hopkins Medicine Institutional Review Board (IRB). Specifically, raw plane wave radio frequency channel data were acquired with an Alpinion ECUBE-12R research ultrasound scanner connected to either an L8-17 or an L3-8 linear array ultrasound transducer, with 75 plane wave transmissions including angles ranging −16° to 16°, or with 73 plane wave transmissions including angles ranging −8° to 8°. The sampling frequency was 40 MHz. Additional details and acquisition parameters are reported in Table I and in the Appendix.

#### 3) Additional Dataset Contributions:

A subset of contributed data not selected by the CUBDL organizers for evaluation of Task 1 are included in the open dataset [16] published with this article for potential future training of new networks. There were various reasons these data were not selected for Task 1, including a desire to maintain sufficient variability, to avoid dominance of data from one group, phantom, subject, or system setting, and to exclude data acquired with focused transmissions for the unattempted Task 3. These additional data consisted of 544 image acquisition sequences (including 529 acquired with plane wave transmissions and 15 acquired with focused transmissions), contributed from MYO, UFL, TSH, EUT, INS, OSL, and JHU. Image targets included simulated structures, experimental phantoms, and *in vivo* heart, breast, brachioradialis, and carotid. The *in vivo* data from JHU and OSL were acquired after informed consent and IRB approval, as noted in [30] and [31], respectively. The *in vivo* data from TSH were acquired after informed consent and ethics approval. The phantom data consisted of 12 different phantoms from six manufacturers including: 1) CIRS models 040, 049, 054GS, 050, 059, and ATS549; 2) GAMMEX models 403 and 410 SCG; 3) NPL Thermal Test Phantom; 4) CAE Blue Phantom Elastography Breast Model; 5) True Phantom Solutions Brain Phantom; and (6) Dansk Phantom Service Model 453. One of these phantom acquisitions (i.e., from MYO) included a layer of *ex vivo* porcine abdominal tissue to introduce acoustic clutter. These data were acquired with 4 ultrasound scanner models (i.e., Verasonics Vantage 256, Verasonics Vantage 128, Alpinion ECUBE12-R, and ULA-OP 256) and 11 ultrasound transducer models. The acquisition center frequencies ranged from 2.97 to 12.5 MHz. The sampling frequencies for this dataset ranged from 6.25 to 100 MHz, representing a larger range than that reported in Section III-A1. The ultrasound transducers consisted of linear and phased arrays. Additional details and acquisition parameters are reported in the Appendix.

#### 4) Sound Speed Correction Applied to Phantom Data:

In ultrasound imaging, the sound speed is often assumed to be globally constant at 1540 m/s when computing focusing time delays. However, the true sound speed often differs from this assumption and depends on specific properties of the underlying tissue. For example, the sound speed of fat is lower than that of liver or muscle [32], [33], and the sound speeds of calibrated tissue-mimicking phantoms are known to vary with the ambient temperature [34]. Improper focusing can degrade image quality, lead to worsened metrics such as contrast and point target resolution, and cause incorrect positioning of image targets. As CUBDL evaluation heavily depended on these and other image quality parameters [20], the CUBDL organizers applied an initial sound speed correction to the contributed test data.

Sound speed correction is an active area of research with many proposed figures of merit, including speckle brightness [35], [36], coherence factor [37], among many others [38]–[41]. Considering this multitude of validated options, the CUBDL organizers selected speckle brightness maximization for its simplicity and its wide acceptance as a criterion of focusing quality [35]. More specifically, the correct global sound speed was selected as the one that maximized the average brightness in a homogeneous region of speckle [36].

PyTorch [42] provides a convenient differentiable framework to perform per-image optimization. Using the same PyTorch implementation of delay-and-sum (DAS) plane wave beamforming provided to CUBDL participants, the sound speed was adjusted via gradient ascent until speckle brightness was maximized using the Adam optimizer (initial step size: 10m/s, decay rate: 0.9, 30 steps). Despite the use of PyTorch, we emphasize that this sound speed correction did not involve any actual deep learning. Specifically, no parameters were trained, and no future predictions of sound speed were performed. PyTorch was simply used to execute a well-known and previously validated sound speed correction algorithm [36] on a per-image basis.

**Fig. 1.**
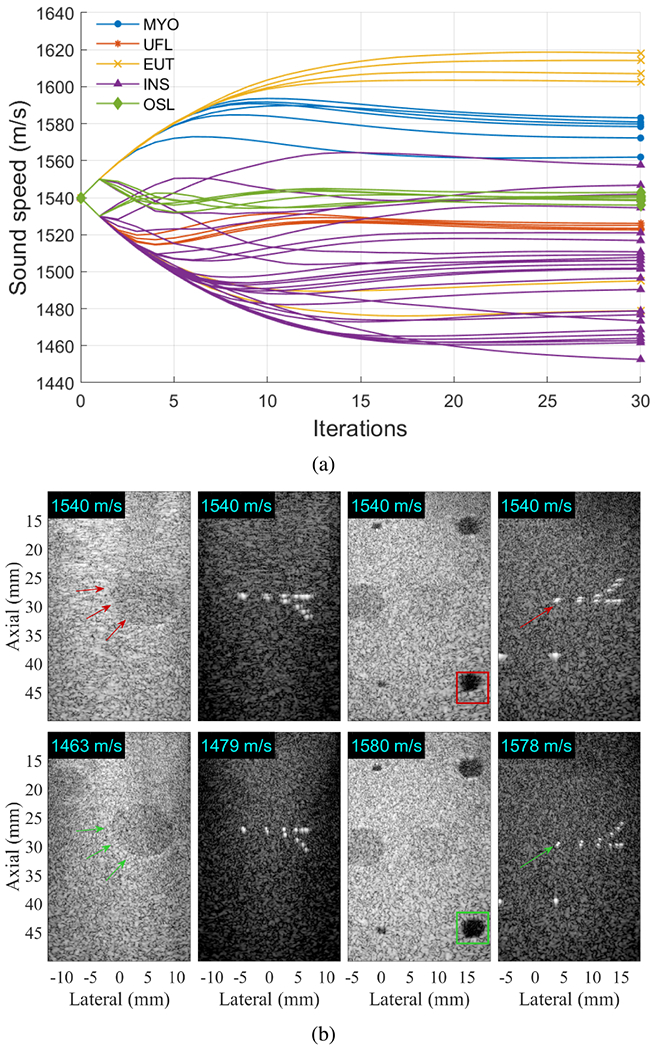
**(a)** Wide range of optimal sound speeds were observed in contributed phantom datasets after 30 iterations of speckle brightness maximization. **(b)** Phantom images from sequences INS023, INS018, MYO001, and MYO003 are shown (from left to right, respectively) before (top) and after (bottom) global sound speed correction, displayed with 50-dB dynamic range. Arrows and boxes highlight locations where lesion boundaries are considerably sharper, point targets are visually more separable, and targets are repositioned to calibrated depths after sound speed correction.

For each of the 49 phantom acquisition sequences acquired using plane wave transmissions (including 19 of the plane wave sequences described in Section III-A1 and 30 of the plane wave sequences described in Section III-A3), the PyTorch DAS beamformer and associated sound speed correction implementation processed the 75 plane-wave raw data in a pixel grid corresponding to a homogeneous region of speckle. Fig. 1(a) shows the sound speeds obtained throughout 30 iterations of speckle brightness maximization for each of the 49 phantom acquisition sequences. After 30 iterations, a wide range of optimal sound speeds were observed across these contributed datasets, ranging from 1453 to 1618 m/s. The corrected value is largely grouped by institution. The two sequences from MYO acquired with the clutter layer (i.e., MYO004 and MYO005) are most deviant in comparison to the remaining sequences from the same institution. The data submitted by INS, which included nine different phantoms, demonstrated the largest variation. The sound speed correction was sensitive to the chosen speckle region for some of these phantoms.

Fig. 1(b) shows example phantom images created from sequences INS023, INS018, MYO001, and MYO003. The sound speed included in the files submitted by data contributors for these four data sequences was 1540 m/s (although the sound speed values provided on data sheets from the associated phantom manufacturers were unlisted, 1540, 1580, and 1580 m/s, respectively). When using 1540 m/s as the sound speed to create images, lesion inclusions and point targets were visible, yet with degraded contrast and resolution, as seen in the top row of Fig. 1(b). However, when the same channel data were used to create images with the corrected sound speeds, this update yielded considerably sharper lesion boundaries, more visually separable point targets, and target repositioning to calibrated depths, as seen in the bottom row of Fig. 1(b). The specific sound speed values used to create the example images in Fig. 1(b) are reported in the top left corner of each image.

This sound speed correction was a necessary step implemented by the CUBDL organizers to enable fair evaluations, and the corrected sound speed values were not shared with the participants while the challenge was open. The corrected sound speeds for the 49 phantoms are now available with the CUBDL evaluation code [15] and associated datasets [16]. During the evaluation process, the corrected sound speeds were provided as inputs to the networks submitted by participants and were used to calculate the image quality metrics reported throughout the article.

#### 5) Summary of Compiled Open Datasets:

In summary, the complete CUBDL + post-CUBDL datasets released with this publication contain a total of 576 acquisition sequences with the following breakdown: 1) 49 experimental phantom data sequences acquired with plane wave transmissions; 2) 11 *in vivo* data sequences from the breast of six patients, each acquired with plane wave transmissions; 3) 500 *in vivo* data sequences from the brachioradialis of a healthy volunteer, each acquired with plane wave transmissions; 4) six experimental phantom data sequences acquired with focused transmissions; 5) eight *in vivo* data sequences comprising the carotid artery of a healthy volunteer, the heart of two healthy volunteers, and the breast of two patients, each acquired with focused transmissions; and 6) 2 Field II [43], [44] simulations.

The phantom data consisted of a total of 13 different phantoms from six manufacturers, including: 1) CIRS models 040, 049, 054GS, 050, 059, and ATS549; 2) GAMMEX models 404GSLE, 403, and 410 SCG; 3) NPL Thermal Test Phantom; 4) CAE Blue Phantom Elastography Breast Model; 5) True Phantom Solutions Brain Phantom; and 6) Dansk Phantom Service Model 453. Three of the phantom acquisitions (i.e., from MYO) included a layer of *ex vivo* porcine abdominal tissue to introduce acoustic clutter.

This wide range of channel data was acquired with four ultrasound scanner models and 11 ultrasound transducer models. The acquisition center frequencies ranged from 2.97 to 12.5 MHz. The sampling frequencies ranged from 6.25 to 100 MHz. The ultrasound transducers consisted of linear and phased arrays. These data were provided by seven groups total: 1) MYO; 2) UFL; 3) EUT; 4) INS; 5) OSL; 6) TSH; and 7) JHU. More details about these data are available in Tables IV through X in the Appendix.

We additionally provide sound speeds for the 49 phantom acquisition sequences acquired using plane wave transmissions with the released datasets [16], including corrected sounds speeds from the procedure described in Section III-A4, sound speeds submitted by data contributors, and sound speeds reported in publicly available datasheets from phantom manufacturers for comparison.

### B. Top Challenge Submissions

#### 1) Rothlübbers et al. [28]:

A fully convolutional network was submitted by Rothlübbers *et al.* [28], and the following summary includes details that are not available in [28]. The network had four layers. The network input was time-delayed, magnitude-normalized, complex-valued data from the 0° plane wave transmission angle. The network output was a real-valued (scalar) weighting factor for each reconstructed pixel. The network was designed to model the united sign coherence factor (USCF) [45] by computing pixel-wise weighting. The final pixel values were obtained by multiplying the unweighted sum absolute pixel values by the network output pixel weights, followed by log compression and a correction for the maximum value.

Batch normalization and ReLU activation followed each convolution. To account for memory limitations during training and inference, a patch-based approach was used, dividing input and target data into patches of size 200 × 200. Convolutions were applied in the channel domain only, resulting in individually processed pixels. This network used an Adam optimizer with a learning rate decay of 0.1 every five epochs, and it was trained for 15 epochs. The loss was computed as a linear combination of mean-squared error (MSE) and multiscale structural similarity (MS-SSIM) [46] loss on the log-compressed, normalized final images. The trained network weights are available with the evaluation code provided with this publication.

The training data consisted of 107 US raw datasets of a phantom (Model 054GS, CIRS, Norfolk, VA, USA), acquired with multiple angles using a 128-element linear array transducer (DiPhAS, Fraunhofer IBMT, Sankt Ingbert, Germany) operating at 4 MHz. High-quality target images were reconstructed using multi-angle USCF imaging [45], using data from seven plane wave angles. The reconstruction grid was chosen with an equidistant isotropic pixel spacing of a third of the wavelength and positioned such that artifact-prone areas such as, for example, near the transducer were excluded. The publicly available PICMUS dataset [21] was used to test the model prior to submitting it to the challenge.

#### 2) Goudarzi et al. [29]:

The details in the following summary parallel the details provided in Section III-B1 if direct comparison between the two networks is desired, and this summary includes additional details that are not available in [29]. The submission by Goudarzi *et al.* [29] used the MobileNetV2 [47] architecture, which consists of depth-wise separable convolution building blocks, linear bottlenecks between layers, and shortcut connections between the bottlenecks. The network input was a 2 × *m* × *n* matrix in which first the two channels were the real and imaginary parts of in-phase and quadrature (IQ) data, *n* is the number of channels, and *m* is the length of the window considered for temporal averaging to preserve the speckle statistics. The network output was a two-dimensional (2-D) vector containing real and imaginary parts of the beamformed data. The network was designed to estimate and apply an apodization window to the input IQ channel data for minimum variance beamforming [48]. The output IQ data were then envelope-detected and log-compressed to obtain the final B-mode ultrasound image. This network used an AdamW optimizer [49] with default parameters (i.e., *β*_1_ = 0.9 and *β*_2_ = 0.999). The loss was computed as the L1-norm between the network output and the IQ pixel values obtained using the minimum variance beamformer. The trained network weights are available with the evaluation code provided with this publication.

The training data for this network consisted of the publicly available plane wave and focused transmission datasets available in the Ultrasound Toolbox [21], [31], [50]. This toolbox contains replicates of data sequences OSL008-OSL009 [51], OSL011-OSL014 [31], and OSL015 [52] in a different file format. Plane wave datasets from this toolbox were acquired with a Verasonics Vantage 256 scanner and L11-4v probe (phantom and *in vivo* data), an Alpinion E-Cube12R scanner and L3-8 probe (phantom data), and Field II simulated data. Focused imaging datasets were acquired with a Verasonics Vantage 256 scanner connected to a P4-2v probe and an Alpinion E-Cube12R scanner connected to a L3-8 probe. Although up to 75 plane waves were available, images were reconstructed from data received after only a single 0° plane wave transmission as the ground-truth output images during training, because the objective when implementing this network was to mimic minimum variance beamforming [48] (which is a real mapping function on the channel data).

### C. Evaluation Metrics

#### 1) Local Image Quality Metric:

Contrast, contrast-to-noise ratio (CNR), generalized contrast-to-noise ratio (gCNR) [51], [53], and signal-to-noise ratio (SNR) within selected regions of interest (ROIs) were measured on the envelope of beamformed images from CUBDL Task 1 data as follows:

(1) $$\text{Contrast}=20\;\log_{10}\left(\frac{\mu_{1}}{\mu_{2}}\right)$$

(2) $$\text{CNR}=\frac{\mu_{1}-\mu_{2}}{\sqrt{\sigma_{1}^{2}+\sigma_{2}^{2}}}$$

(3) $$\text{gCNR}=1-\underset{x}{\sum }\underset{x}{\min}\left\{f_{1}(x),f_{2}(x)\right\}$$

(4) $$\text{SNR}=\frac{\mu_{o}}{\sigma_{o}}$$

where *μ_i_, σ_i_*, and *f_i_* represent the mean, standard deviation, and histogram, respectively, of ROI *i*. The gCNR metric was calculated with 256 bins. The SNR metric was used to measure speckle preservation with the ground truth derived from the 75 plane wave image. In addition to the above metrics, the axial and lateral full-width at half maximum (FWHM) of point targets was calculated to determine resolution. Details surrounding the specific ROIs chosen for each test case are available in in the Appendix and in our public evaluation code [15]. This code will further include routines for evaluating speckle pattern autocorrelations for resolution measurements.

#### 2) Global Image Quality Metrics:

The more global *ℓ*_1_ loss, *ℓ*_2_ loss, peak signal-to-noise ratio (PSNR), and normalized cross correlation (*ρ*) metrics were computed from CUBDL Task 1 data for image-to-image comparisons between network-produced envelope images and the corresponding 75 (or 31 for sequence TSH002) plane wave envelope image

(5) $$\ell_{1}=\frac{1}{N}\overset{N}{\underset{n=1}{\sum }}\left|x_{n}-y_{n}\right|$$

(6) $$\ell_{2}=\sqrt{\frac{1}{N}\overset{N}{\underset{n=1}{\sum }}{\left|x_{n}-y_{n}\right|}^{2}}$$

(7) $$\mathrm{PSNR}=20\;\log_{10}\frac{\text{Dynamic Range}}{\sqrt{\frac{1}{N}{\sum_{n=1}^{N}\left|x_{n}-y_{n}\right|}^{2}}}$$

(8) $$\rho =\frac{\sum_{n}\left(x_{n}-\mu_{x}\right)\left(y_{n}-\mu_{y}\right)}{\sqrt{\left(\sum_{n}{\left|x_{n}-\mu_{x}\right|}^{2}\right)\left(\sum_{n}{\left|y_{n}-\mu_{y}\right|}^{2}\right)}}$$

where *x* and *y* denote the two images being compared, each containing *N* pixels. The image comparisons were performed using pixels within the dynamic range of −40 to 0 dB with respect to the maximum pixel value of the ground truth image to avoid overemphasis of small magnitude differences and to avoid penalizing networks that do not reproduce the acoustic clutter that resides at magnitudes less than −40 dB [54]. In addition, both the *ℓ*_1_ and *ℓ*_2_ losses were computed for images on a linear scale (i.e., before log compression, denoted as *ℓ*_1_ and *ℓ*_2_, respectively) and on a log-compressed scale (i.e., after log compression, denoted as *ℓ*_1_-log and *ℓ*_2_-log, respectively). The linear scale is expected to be more sensitive to high-amplitude variations (e.g., edges), whereas the log scale is more sensitive to low-amplitude variations (e.g., speckle). These metrics were computed on images that were normalized to minimize the achievable loss, as described in the Appendix of [11]. Overall, these global image quality metrics provide quantitative information that is not subject to the region selection requirement of the local metrics.

#### 3) Network Complexity:

Network complexity was determined by the number of learnable parameters in each submitted model. Although the CUBDL organizers initially intended to include network speed as an additional parameter to assess network complexity, there was greater variety in the “preprocessing” prior to entering the network than anticipated, which complicated fair comparisons of network speed. For example, the submission by Goudarzi *et al.* [29] needed the data to be reshaped and reformatted into 1-D kernels along the axial dimension, all of which was implemented outside of PyTorch, whereas the two submissions from the runners up [19], [27] needed a 2-D image interpolation in numpy. For this reason, the CUBDL organizers only considered network size, as each participant successfully addressed the proposed task which purposefully did not specify which component of the image formation process had to be learned.

### D. Scoring and Ranking

The four CUBDL participants were evaluated using the test data described in Section III-A1 and received a rank based on each quantitative network performance described in Section III-C. These rankings were grouped into two categories: 1) image quality and 2) network complexity, considering that participants were challenged to balance both image quality and display frame rates. We averaged the ranks of the metrics obtained by each participant within these two groups. The average rank from each group was summed. This scoring system is represented mathematically as follows:

(9) $$\text{Final Score}=\frac{\sum \;\text{image quality metric rankings}}{T_{\mathrm{IQ}}}+\frac{\sum \;\text{Network complexity metric rankings}}{T_{\mathrm{NC}}}$$

where *T*_IQ_ and *T*_NC_ are the total numbers of image quality metric rankings and network complexity rankings, respectively.

### E. Post-CUBDL Analyses

To improve the robustness of our evaluation methods and to ensure the robustness of our open-source code, two additional analyses were implemented. First, a two-sample, two-tailed *t*-test with a 5% significance level was performed to determine the statistical significance of differences among the top two submitted networks described in Section III-B when evaluating the global image-to-image metrics described in Section III-C2. Second, the test data were evaluated by an independent user (A.W.) after the challenge was closed to new submissions. This independent user provided feedback to increase compatibility of the evaluation framework and also used this framework to evaluate the submitted networks with the post-CUBDL data described in Section III-A2.

## IV. Results

### A. Network Performance Evaluation

#### 1) Baseline Evaluation:

**Fig. 2.**
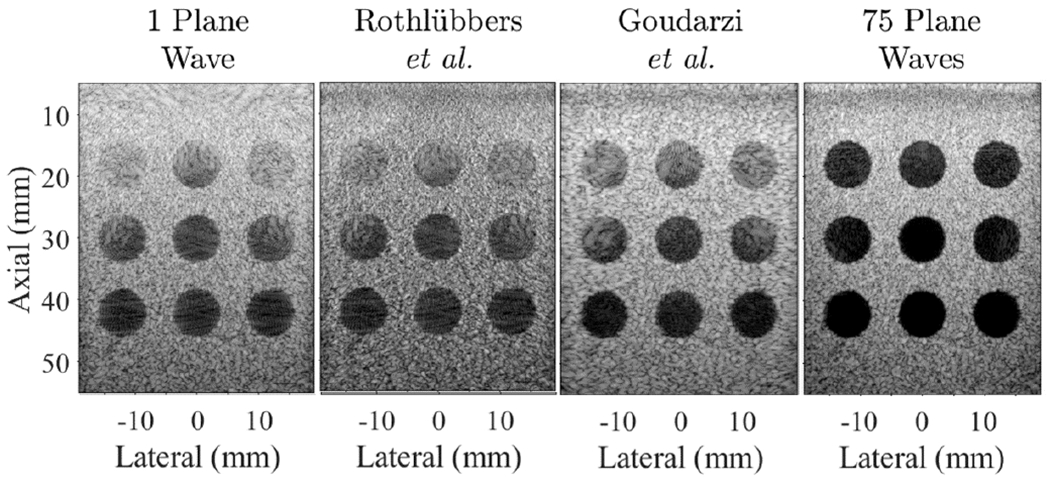
Results from the baseline PICMUS dataset [21] of simulated anechoic lesions, displayed with 60-dB dynamic range.

To ensure that participant submissions from different deep learning frameworks (e.g., Tensor-Flow and PyTorch) were properly loaded into our evaluation code, we used a baseline evaluation on the publicly available PICMUS data [21], as shown in Fig. 2. All submissions were confirmed to produce images morphologically similar to the single and 75 plane wave images before proceeding with the evaluation [14].

#### 2) Lesion Targets:

**Fig. 3.**
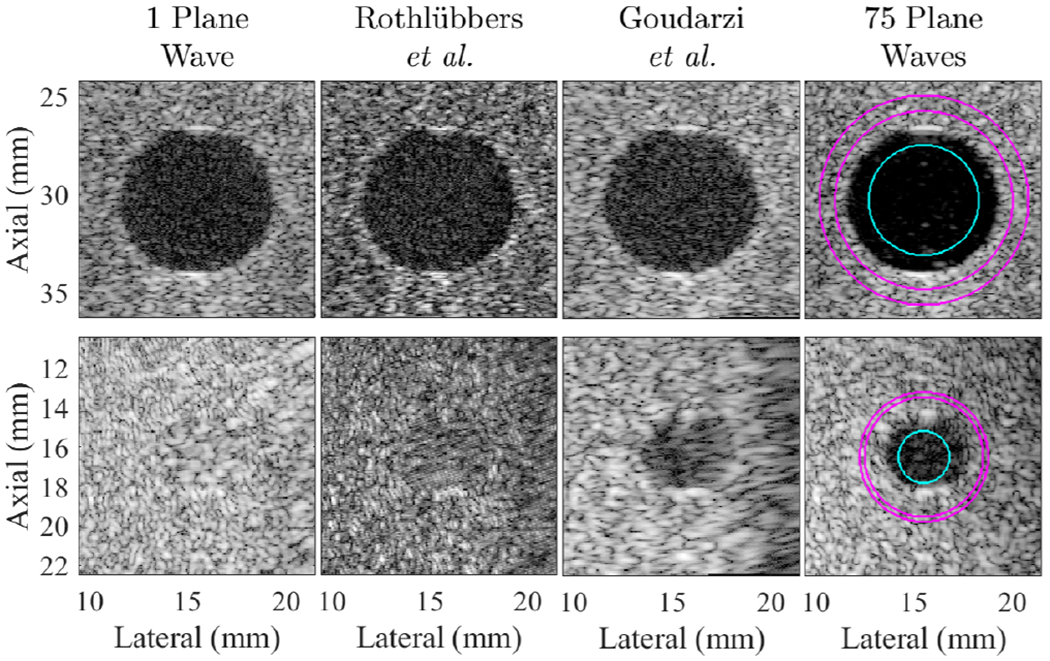
Example results from the lesion test set, taken from dataset sequences UFL001 (top) and MYO001 (bottom), displayed with 40-dB dynamic range. The ROIs used for image quality metrics are overlaid on the 75 plane wave images, which served as the ground truth.

Fig. 3 shows two lesion examples from dataset sequences UFL001 and MYO001. Each column contains the results produced by the top two submitted networks, with the single and 75 plane transmission results shown for comparison on the far left and far right, respectively. Both submitted networks successfully created the larger lesion shown on the top of Fig. 3 with contrast, CNR, and gCNR of −15.29 dB, 1.40, and 0.86, respectively, for the network submitted by Goudarzi *et al.* [29] and −11.13 dB, 0.84, and 0.62, respectively, for the network submitted by Rothlübbers *et al.* [28]. The corresponding contrast, CNR, and gCNR of the single and 75 plane wave results for this lesion were −14.65 dB, 1.38, and 0.84, and −25.84 dB, 1.58, and 0.96, respectively.

For the smaller lesion on the bottom of Fig. 3, the network submitted by Goudarzi *et al.* [29] produced the only successful result for this lesion (contrast, CNR, and gCNR of −12.38 dB, 1.44, and 0.82, respectively), which is remarkable considering that a single plane wave transmission was not sufficient to visualize this lesion with the traditional beamforming methods. The corresponding contrast, CNR, and gCNR of the single and 75 plane wave results for this lesion were −1.16 dB, 0.19, and 0.24 and −17.25, 1.35, and 0.88, respectively.

The quantitative results for lesion visibility were measured for a total of eight ROIs from the following seven sequences: UFL001, UFL005 (two independent ROIs from this sequence), OSL007, MYO001, MYO004, INS008, and INS021. The mean ± standard deviation of the contrast, CNR, and gCNR for the eight ROIs is reported in Table II. No participants achieved greater lesion visibility or detectability than that obtained with 75 plane wave transmissions. Goudarzi *et al.* [29] achieved the best lesion visibility with the least variance overall and also achieved the closest metrics to the 75 plane wave results, while in some cases exceeding the single plane wave result.

#### 3) Speckle Targets:

**Fig. 4.**
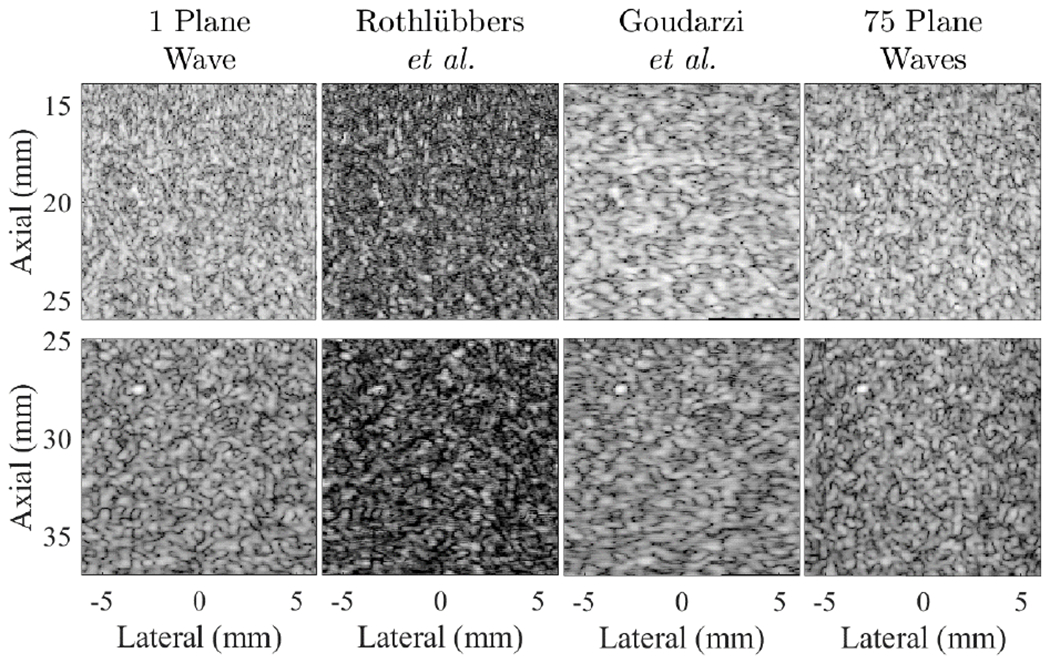
Example results from the speckle test set, taken from dataset sequences MYO002 (top) and INS016 (bottom), displayed with 40-dB dynamic range.

Fig. 4 shows examples from two speckle targets taken from dataset sequences MYO002 and INS016. Most participants achieved results that qualitatively resembled speckle texture. Goudarzi *et al.* [29] best achieved the goal of preserving speckle SNR from both single and 75 plane wave transmissions, when measured from six ROIs total from the following sequences: UFL004, OSL007, MYO002, EUT003, INS004, and INS016. The mean ± standard deviation of speckle SNR within these six ROIs is reported in Table II.

#### 4) Point Targets:

**Fig. 5.**
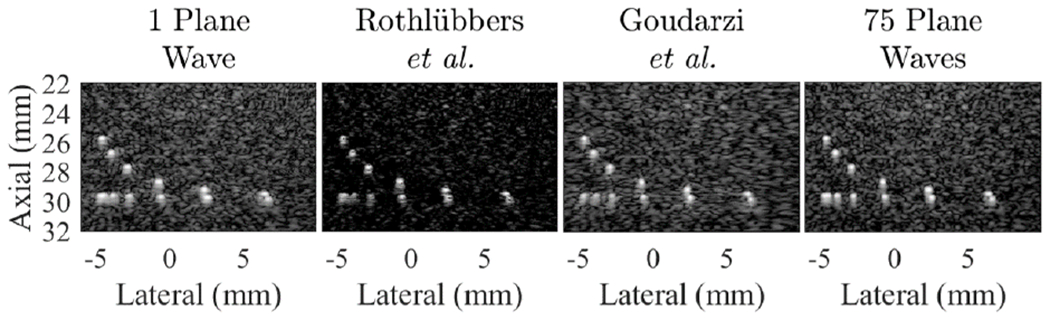
Example results from the point target test set, taken from dataset sequence UFL004, displayed with 40-dB dynamic range.

Fig. 5 shows example results from the point target evaluation, taken from dataset sequence UFL004. There were a total of four ROIs from sequences MYO002, MYO003, UFL002, and UFL004, each containing 3–5 points that were evaluated. The mean ± standard deviation of the lateral and axial resolution of these point targets is reported in Table II as axial and lateral FWHM, respectively. It is remarkable that the networks submitted by Rothlübbers *et al.* [28] and Goudarzi *et al.* [29] achieved better resolution results when compared with standard DAS imaging with both single and 75 plane wave transmissions, considering that the network images were created from channel data acquired after a single plane wave transmission.

#### 5) Image Targets:

**Fig. 6.**
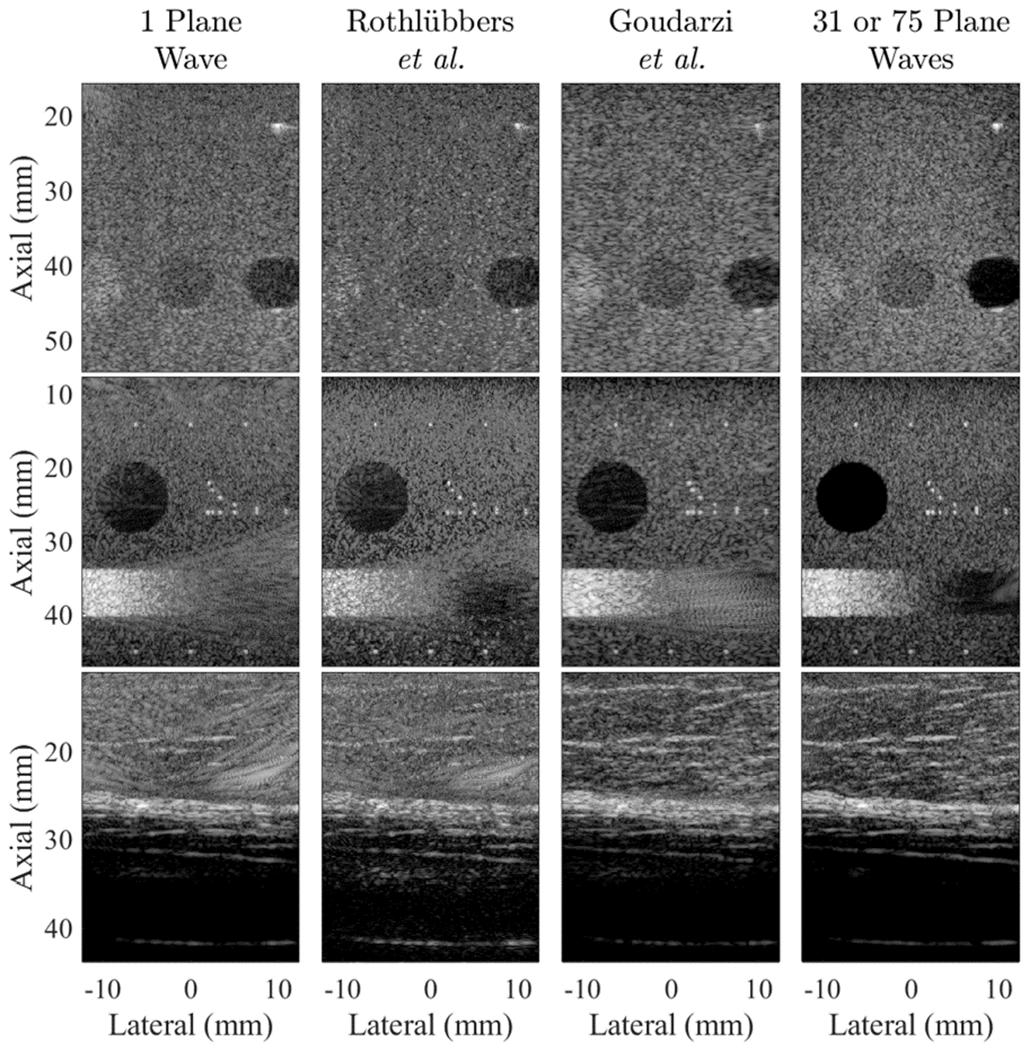
Example results from the image test set, taken from dataset sequences INS008, OSL010, and TSH002, from top to bottom, respectively, displayed with 60-dB dynamic range. The right column shows images created after 31 (TSH002) or 75 (INS008, OSL010) plane wave transmissions.

Fig. 6 shows examples from three image targets. These example images were taken from data sequences OSL010, INS008, and TSH002. Network and single plane wave results were compared with the 75 plane wave images, using the global image-to-image metrics described in Section III-C2 and the 12 images created with data sequences EUT006, INS006, INS008, INS015, INS019, MYO001, MYO002, MYO004, MYO005, OSL010, UFL002, and TSH002. The corresponding image-to-image comparison results are reported in Table III.

Summarizing the results in Table III, there were no statistically significant differences (*p* > 0.05) when comparing the mean *ℓ*_1_, *ℓ*_1_-log, *ℓ*_2_, and PSNR of the networks submitted by Goudarzi *et al.* [29] and Rothlübbers *et al.* [28]. However, there were statistically significant differences (*p* < 0.05) when comparing the mean *ℓ*_2_-log and *ρ* of the networks submitted by Goudarzi *et al.* [29] and Rothlübbers *et al.* [28]. The corresponding image quality metrics obtained when the single plane wave results were compared with the 75 plane wave results from the same datasets are also reported in Table III for additional comparison. With the exception of *ℓ*_1_, these single plane wave results produce the best mean values across the 12 image targets included in the CUBDL evaluation dataset (as indicated by the bold text in Table III), where lower values of *ℓ*_1_, *ℓ*_2_-log, *ℓ*_1_, *ℓ*_2_-log, and higher values of *ρ* and PSNR indicate better matches with the ground-truth 75 plane wave results. Note that for cases with identical mean *ℓ*_1_ and *ℓ*_2_ values in Table III, the corresponding values with the lower mean *ℓ*_1_-log and *ℓ*_2_-log, respectively, were bolded.

#### 6) Rankings:

Based on the first additive term of (9), the images created with 75 plane wave transmissions achieved the highest rank in a majority of cases (i.e., 1), followed by images created with DAS beamforming after a single plane wave transmission, then the network submitted by Goudarzi *et al.* [29], and then the network submitted by Rothlübbers *et al.* [28]. Specifically, the average ranks for image quality [i.e., the first additive term of (9)] were 1.25, 2.58, 2.67, and 3.58, respectively, for the rank order provided in the preceding sentence. Based on the second additive term of (9), Rothlübbers *et al.* [28] submitted a network with significantly less network complexity (i.e., 3059 parameters) when compared with that of Goudarzi *et al.* [29] (i.e., 2226146 parameters), as reported in Table II. Therefore, Rothlübbers *et al.* [28] received a rank of 3 in the network complexity category, followed by Goudarzi *et al.* [29] receiving a rank of 4, considering that both single and 75 plane wave DAS implementations had 0 trainable parameters for this evaluation.

When combining the two additive terms in (9) to determine the winner, Rothlübbers *et al.* [28] and Goudarzi *et al.* [29] achieved final scores of 6.58 and 6.67, respectively, resulting in both being declared by the CUBDL organizers as the challenge winners. This declaration was made because Goudarzi *et al.* [29] achieved both qualitatively and quantitatively high-quality images, and at the same time, it was remarkable that Rothlübbers *et al.* [28] produced a network with significantly low complexity (and generally acceptable image quality with the best lateral and axial resolution overall).

### B. Performance With Post-CUBDL In Vivo Data

**Fig. 7.**
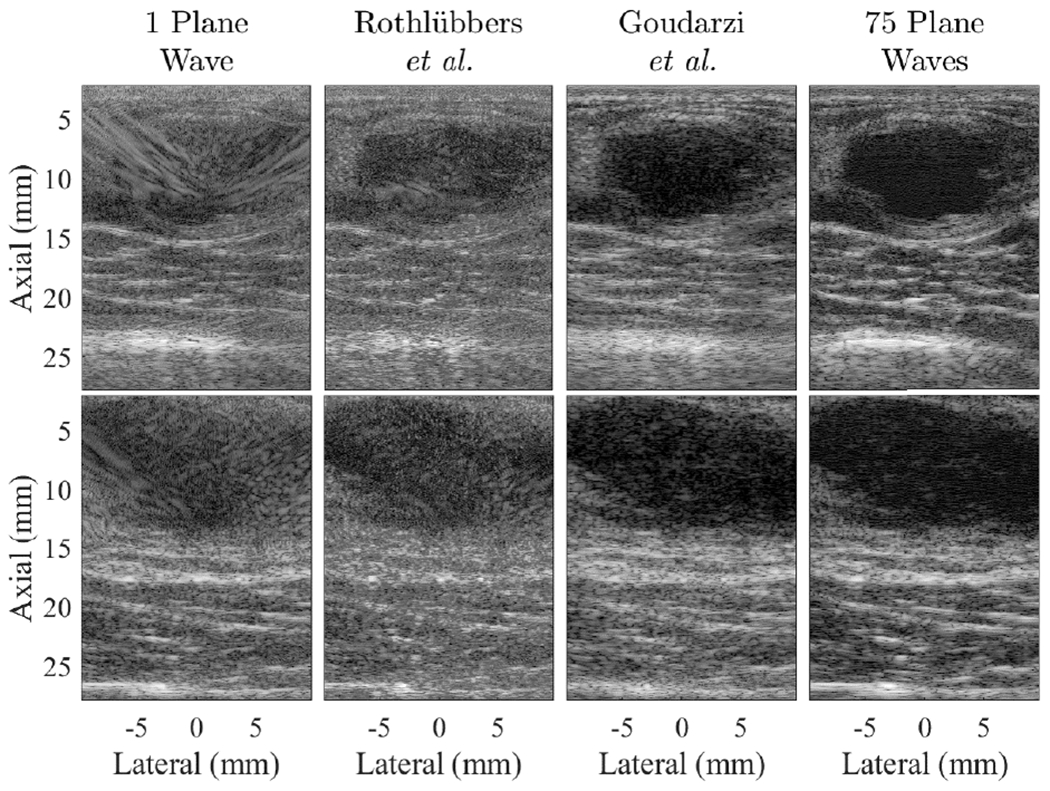
Example results of *in vivo* breast masses from the post-CUBDL test set, taken from sequences JHU028 (top) and JHU030 (bottom), displayed with 60-dB dynamic range.

Fig. 7 shows example *in vivo* breast images from the JHU post-CUBDL dataset described in Section III-A2. These example images were taken from data sequences JHU028 and JHU030. Network and single plane wave results were compared with the 75 plane wave images, using the global image-to-image metrics described in Section III-C2 and the 11 images created with data sequences JHU024 through JHU034. The quantitative image-to-image comparison results for this dataset are reported in Table III.

The framework developed using the initial CUBDL evaluation test set was able to seamlessly incorporate the JHU post-CUBDL *in vivo* breast data, which was not included in the initial test set. Remarkably, the submitted networks also generalized to these new data from a different imaging environment. We observed qualitative differences between the *in vivo* breast images produced by the two submitted networks, and the results in Table III demonstrate that the *ℓ*_2_-log and *ρ* metrics consistently show statistically significant differences across both datasets (i.e., CUBDL and post-CUBDL data). Table III also shows that the network submitted by Goudarzi *et al.* [29] produced consistently better quantitative image-to-image comparisons than the network submitted by Rothlübbers *et al.* [28] across both datasets. In addition, the results obtained when testing this network [29] on the post-CUBDL *in vivo* dataset were consistently better than the results obtained with DAS beamforming of the single plane wave data (as indicated by bold in Table III). For this post-CUBDL dataset, the network submitted by Rothlübbers *et al.* [28] also produced better qualitative results in some cases (e.g., Fig. 7, sequence JHU028) than the corresponding single plane wave result.

## V. Discussion

### A. Overview of Challenge Outcomes

The details described in this article serve the threefold purpose of: 1) summarizing, comparing, and drawing insights from the top CUBDL submissions; 2) providing a detailed description of the evaluation process for future benchmarking of deep neural networks designed to create ultrasound images; and 3) describing the totality of shared evaluation code and datasets for ease of future reproducibility and replication. Our major challenge outcomes include the largest known international database of raw ultrasound channel data, network descriptions and trained network weights from the CUBDL winners, a PyTorch DAS beamformer containing multiple components that can be converted to trainable parameters, a data sheet of phantom sound speeds containing optimal speeds identified by the CUBDL organizers using the PyTorch DAS beamformer, and evaluation code that integrates these major outcomes.

During the evaluation process, the first step to achieving the challenge objectives was to ensure that the image reconstruction process offered a fair comparison despite the wide range of test data from multiple groups. Hence, the development of the neural-network-based PyTorch DAS beamformer and the associated sound speed correction (described in Section III-A4) were key contributions to enable fair comparisons. While these contributions are the first to implement a differential framework for sound speed correction, there are other solutions that implement DAS within a computational framework capable of automatic differentiation (e.g., PyTorch or TensorFlow) [55]–[57].

The results from these sound speed correction contributions (i.e., Fig. 1) support the importance of including a standardized international dataset for deep learning network development (e.g., training, testing, and evaluation). In particular, a dataset containing incorrect sound speeds and limited acquisition parameter variability may not generalize well to data acquired under different conditions. Sound speed differences within groups (e.g., INS) and between groups (e.g., MYO and UFL) that imaged the same phantom models can be attributed to a combination of ambient environment, the presence of an *ex vivo* tissue layer producing acoustic clutter, and phantom construction differences. Considering that many of the observed phantom variations occurred in the absence of tissue layers and the speckle brightness sound speed correction method was previously validated [36], environmental factors (e.g., ambient temperatures, phantom degradation) are considered to be the primary contributor to these observed differences.

### B. Quality of Image Formation With Deep Learning

One of the underlining questions surrounding CUBDL Task 1 was an inquiry regarding the capability of deep learning to create high-quality images after a single plane wave transmission. The image quality summary in Table II indicates three insights and capabilities of deep neural networks implemented for beamforming. First, they are capable of preserving speckle SNR. Second, they may be capable of improving resolution. Third, the submitted networks (which created images after only a single 0° plane wave transmission) were capable of producing better qualitative, quantitative, and lesion detectability results than the single plane wave DAS result in some cases (e.g., Fig. 3 sequence MYO001, Figs. 5–7, Tables II and III). It remains to be determined whether the latter two capabilities can be attributed to the choice of target beamformer (i.e., USCF, minimum variance) during training, as opposed to the function-approximating nature of the deep learning approach itself. In addition, the submitted networks had difficulty obtaining better lesion detectability than the 75 plane wave DAS images. These observations are based on the presented image quality metrics and are supported by the image quality rankings, with associated limitations discussed in more detail in Section V-E. For example, although speckle SNR was quantitatively preserved in some cases (see Table II), it was not always qualitatively preserved (e.g., MYO002 in Fig. 4), when comparing both single plane wave results and network results with the 75 plane wave results.

We also observed that learning a single, intermediate step of the image formation pipeline (as pursued by Goudarzi *et al.* [29] and Rothlübbers *et al.* [28]) represents a more clearly defined transformation for the presented task, as opposed to attempts to learn the entire beamforming process (i.e., the approach taken by [19] and [27], which suffered from overfitting to the training data, as shown in the presentation of all challenge results [14]). To briefly summarize for the context of this article, overfitting manifested as an inability of the submitted networks to detect point targets in the unseen test data, recreate lesions from the unseen test data, or replicate the gradation from light to dark shown in Fig. 6 (sequence OSL010). Instead, the images looked more like the learned tissue texture or lesions in the training images in most cases. The training datasets curated by the respective authors of these two runner-up submissions contained similar data to each corresponding testing dataset, and the ability of the resulting networks to produce acceptable results on these similar data is evident in the articles describing the associated submissions [19], [27]. These two networks were also able to successfully reproduce the results shown in Fig. 2, as presented in [14], likely because the PICMUS data were included in the training sets of both networks. These observations highlight successes that can be achieved when the training and testing sets are similar, with a discussion of the overfitting implications available in Section V-D in the context of future recommendations.

### C. Speed of Image Formation

It is promising that the improvements to image quality were obtained with network complexity levels that indicate a possibility for improved speed compared with the traditional implementations of advanced beamforming approaches. Although the CUBDL organizers did not specify which parts of the image formation task had to be learned, participants were aware that they would be evaluated based on the number of trainable parameters within submitted networks. Participants therefore had the freedom to decide whether they used a network to replace the delay step, the sum step, both steps, a post-processing filter step, and so on. This freedom somewhat complicated the network complexity evaluation, considering that a neural network has the potential to outperform deterministic DAS at the cost of adding parameters that need to be trained. If a network replaces both “delay” and “sum,” it might be more complex than a network that replaces just the “sum,” but it may also rely on fewer assumptions and thus would have greater potential to give better results. This tradeoff was considered to be captured sufficiently by counting the number of trainable parameters.

It is possible that an additional dimension of complexity could have been included by adding a metric for “number of processing components replaced”. However, this addition is not trivial to implement, considering the wide range of possible steps outside of basic DAS beamforming. While different network architectures with the same number of parameters may have different inference speeds, oftentimes this difference in speed is due to nuanced hardware- and software-specific optimizations. It is also possible that we could have estimated the number of floating point operations (FLOPs), but there are multiple methods and implementations for estimation with no clear standard. These considerations point to the additional importance of learning from these CUBDL challenge results to produce updated guidelines if a similar undertaking is attempted in the future.

### D. Future Recommendations

The observations summarized in Section V-B highlight the importance of a truly blind test set to detect and prevent possible overfitting, which is a secondary outcome of the CUBDL challenge (i.e., the newly available database [16] and evaluation code [15] being released with the publication of this article). Regarding the choice not to provide training data for CUBDL participants, the intent was to create a challenge that represented the current state of ultrasound imaging research. In particular, prior to the dataset contribution described in this article, there was no previously curated standard training set specifically catered to deep learning tasks for ultrasound image formation. Instead, the CUBDL organizers encouraged participants to make intellectual contributions on both the choice of architecture and training data, rather than to find a single optimal architecture for a given training dataset.

Now that the challenge is completed, we propose the use of the datasets described in Sections III-A1 and III-A2 as testing data (with caution to avoid overfitting if the dataset described in Section III-A3 is also used for training). Similarly, we propose the use of the top CUBDL challenge submissions as benchmarks for future work in this research domain. For example, related work on the topic of CUBDL Task 1 published after the close of the challenge [58]–[62] can potentially be reevaluated and compared using the CUBDL data [16] and code [15] released with this publication, with no alterations to network training weights and with attention to the overfitting challenges experienced by the runners-up, particularly when considering that some of these methods were similarly trained and tested with the PICMUS data. We are essentially challenging the community to achieve better results than the CUBDL submissions and to make more headway toward explaining why the results are better based on the open research questions articulated in this discussion of challenge results. We also welcome new contributions to the field of ultrasound beamforming with deep learning (and possibly beamforming without deep learning) based on new ideas conceived while reading this article and while working with our open source data and code. The shared datasets and code provide a major step toward making meaningful comparisons in future work.

### E. Limitations and Additional Considerations

Three possible factors that may be viewed as limitations of administering CUBDL include the novelty of the sound speed correction approach, the subjectivity of decisions surrounding the ranking method (which is arguably a limitation of any challenge [63]), and the diversity of the datasets.

First, sound speed optimization is not widely used, and its implementation in a deep learning framework (i.e., PyTorch) may be considered novel and thus inappropriate to introduce in a challenge. The decision to include sound speed correction was threefold: 1) correction was critically needed to enable fair contrast and resolution evaluations in the challenge [see Fig. 1(b)]; 2) the sound speed correction algorithm itself was based on a well-established criterion [35] (see Section III-A4); and 3) the specific implementation of the algorithm (e.g., traditional brute-force search versus PyTorch optimization) does not affect the final result. Thus, the PyTorch-optimized sound speeds were included in the challenge. A potential limitation of this work is that further validation of the sound speed correction was considered out of scope and thus not performed. However, we note that this correction was intended solely as a coarse improvement over assuming 1540 m/s rather than as a perfect sound speed correction, which cannot be achieved by any assumed global sound speed due to the heterogeneity of real targets.

Second, CUBDL placed an equal weighting on the objectives of image quality and network complexity, which may be considered arbitrary and subjective. Indeed, establishing absolute rankings in any multi-objective task is non-trivial because it is possible to be optimal in one metric but sub-optimal in another, that is, to be “Pareto-optimal.” In such a system (known as a partial order), the only way for one method to be definitively superior is to dominate all others in all categories. Although a worthy goal, such an optimum often does not exist and thus cannot be relied upon for challenge administration. Furthermore, it is important to recognize that each image quality metric alone measures a narrow aspect of image quality, such that optimality in that individual metric may not correspond to perceived image quality. For example, speckle SNR measures only first-order statistics and does not capture second-order statistics of speckle structures, making it a poor indicator of image quality alone; however, the (*ℓ*_1_ and (*ℓ*_2_ losses provide complementary spatial information. To reliably obtain a final rank (a total order) from multiple independent categories, one must necessarily apply subjective opinions as to the importance and weighting of each category.

Our chosen approach (detailed in Section III-D) effectively placed less weight on any individual image quality metric and greater weight on overall network complexity. The CUBDL organizers made a conscious decision to emphasize network complexity as a distinguishing factor in anticipation of numerous submissions with similar imaging performance, considering that ultrasound beamforming needs to be real-time for practical application and implementation. It is possible that a low-complexity network producing poor image quality would have achieved a good score with the chosen approach. In particular, according to the image quality rankings reported in Section IV-A6, the current approach favored single plane wave DAS over the submitted networks due to a combination of the better rank performance of the global metrics (summarized in Table III for Task 1 data) and the absence of trainable parameters with traditional DAS beamforming (despite the better performance of the networks over single plane wave DAS images with regard to local metrics, as summarized in Table II). Other weightings are equally subjective, thus equally valid, and may result in different final rankings. The CUBDL organizers are making all data and evaluation scripts available with the publication of this article, leaving room for others to implement alternative analyses if desired.

Finally, while the diversity of the test datasets is viewed as a strength with regard to assessing network generalizability (as discussed in Section V-A) and with regard to detecting possible overfitting to training data (as discussed in Section V-D), this diversity may have also been viewed as a barrier for entry into the challenge. In particular, variations in multiple parameters that are routinely modified to achieve a desired ultrasound image were advertised (e.g., variations in transducers, ultrasound systems, system operators, plane wave angles, center frequencies, sampling frequencies, bandwidths, transducer properties, image depths, and imaging targets), with example images provided in the CUBDL Data Guide [14] and replicated in [20]. Although the PICMUS data were advertised and available, the PICMUS data did not contain this wide range of varying parameters. Concerns regarding limited knowledge of either beamforming or deep learning may have also been a barrier to entry. However, the CUBDL participants reported experience levels with beamforming and deep learning that ranged from novice to expert [14], [20].

## VI. Conclusion

This article summarizes the results of the CUBDL challenge, as well as the detailed evaluation process implemented by the CUBDL organizers and associated insights gained from the evaluation process and challenge results. Evaluation was further analyzed independently, and the associated evaluation code and datasets are newly released with this publication. The open datasets include the CUBDL test data, additional *in vivo* breast data included after the close of the challenge, and a subset of internationally crowd-sourced data that were not used for evaluation, but may potentially be used for future network training and comparison to the results presented within this article. The complete dataset includes 576 image acquisition sequences and represents the largest known open international database of ultrasound channel data. We are releasing this combination of CUBDL results, evaluation code, and open datatasets to our community in efforts to help standardize and accelerate research at the intersection of ultrasound beamforming and deep learning. We additionally propose the use of the top CUBDL challenge submissions as benchmarks for future work in this research domain, and we share the totality of released resources to enable meaningful comparisons of future methods.

## Figures and Tables

**TABLE I:** Summary of CUBDL Task 1 Test Data and Additional Post-CUBDL Evaluation Data

Institution	Total # of Sequences	Data Type	US Scanner	Transducer	Center Frequency (MHz)	Sampling Frequency (MHz)	Number of Plane Waves	Image Depth (mm)	Reference
TSH	1	*In Vivo*	Verasonics Vantage 256	L10-5	7.5	6.25	31	40	[17]
MYO	5	Phantom	Verasonics Vantage 256	L11-4v	6.25	28.96	75	63.8	–
UFL	4	Phantom	ULA-OP256	Esaote LA533	8	78.125	75	50	–
EUT	2	Phantom	Verasonics Vantage 128	Phillips S5-1	3.1	12.5	75	100	–
INS	3	Phantom	Verasonics Vantage 256	L7-4	5.2	20.8	75	56.7	–
INS	4	Phantom	Verasonics Vantage 256	L12-5	7.8	31.25	75	56.7	–
OSL	1	Phantom	Verasonics Vantage 256	L7-4	5.2	20.8	75	50	–
OSL	1	Simulation	Field II	N/A	5.13	20.8	75	50	[18]
JHU*	9	*In Vivo*	Alpinion ECUBE-12R	L8-17	12.5	40	73-75	30	[19]
JHU*	2	*In Vivo*	Alpinion ECUBE-12R	L3-8	5.5	40	75	30	[19]

* Post-CUBDL evaluation data

**TABLE II:** Summary of Local Image Quality Comparisons and Network Complexity, Obtained With [28] and [29] Evaluated on Single Plane Wave Data

	Contrast (dB)	CNR	gCNR	Speckle SNR	Lateral FWHM (mm)	Axial FWHM (mm)	Number of Learned Parameters
Single Plane Wave	−10.50 ± 5.40	1.05 ± 0.44	0.67 ± 0.23	1.85 ± 0.04	0.42 ± 0.07	0.36 ± 0.15	0
Rothlübbers *et al.* [28]	−9.82 ± 4.93	0.67 ± 0.26	0.53 ± 0.20	1.19 ± 0.16	0.32 ± 0.10	0.33 ± 0.13	3,059
Goudarzi *et al.* [29]	−13.77 ± 3.07	1.34 ± 0.25	0.81 ± 0.09	1.81 ± 0.06	0.34 ± 0.10	0.36 ± 0.15	2,226,146
75 Plane Waves	−24.71 ± 5.55	1.53 ± 0.32	0.95 ± 0.05	1.81 ± 0.08	0.36 ± 0.07	0.34 ± 0.14	0

**TABLE III:** Summary of Global Image Quality Comparisons (With Bold Indicating the Best Mean Results for Each Metric)

		*ℓ*_1_	*ℓ*_1_-log	*ℓ*_2_	*ℓ*_2_-log	*ρ*	PSNR
**CUBDL Task 1 Image Targets**	Rothlübbers *et al.*	0.05 ± 0.05	0.48 ± 0.13	0.06 ± 0.05	0.71 ± 0.19*	0.83 ± 0.04*	29.03 ± 5.96
	Goudarzi *et al.*	0.03 ± 0.03	0.42 ± 0.08	0.05 ± 0.04	0.56 ± 0.11*	0.91 ± 0.04*	29.10 ± 6.76
	Single Plane Wave	**0.03 ± 0.03**	**0.39 ± 0.09**	**0.04 ± 0.04**	**0.53 ± 0.11**	**0.93 ± 0.03**	**30.36 ± 7.43**

**Post-CUBDL *In Vivo* Data**	Rothlübbers *et al.*	0.04 ± 0.01	0.62 ± 0.06*	0.06 ± 0.01*	0.80 ± 0.08*	0.77 ± 0.03*	25.90 ± 2.06
	Goudarzi *et al.*	**0.03 ± 0.01**	**0.54 ± 0.04** *	**0.05 ± 0.01** *	**0.71 ± 0.05** *	**0.84 ± 0.02** *	26.02 ± 1.65
	Single Plane Wave	0.03 ± 0.01	0.61 ± 0.10	0.05 ± 0.01	0.77 ± 0.12	0.84 ± 0.05	**26.12 ± 2.40**

* indicates statistically significant differences (*p*<0.05) between Rothlübbers *et al.* [28] and Goudarzi *et al.* [29]

**Fig. 8.**
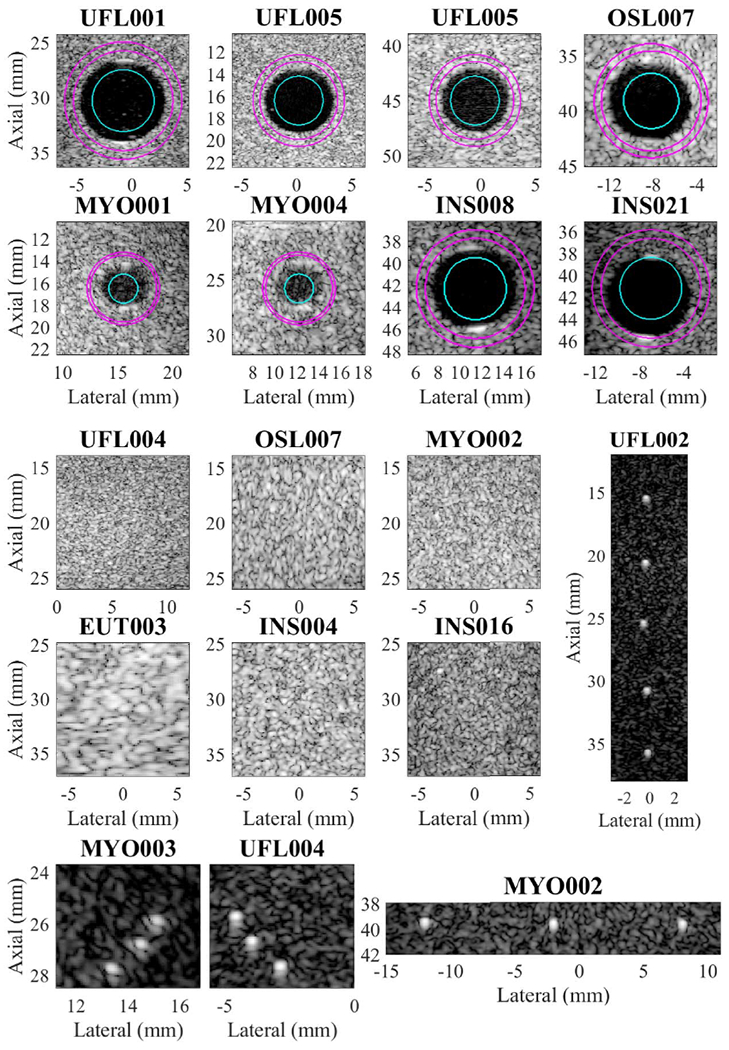
Images created with 75 plane wave transmissions showing the ROIs used to measure contrast, CNR, gCNR (where ROI_1_ is indicated by the inner circle and ROI_2_ is indicated by the concentric outer circles), speckle SNR, and point target FWHM. These images are displayed with 40-dB dynamic range.

## Appendix

The Appendix includes seven tables describing contributed ultrasound channel data (see Tables IV–X), followed by the 18 ROIs used to calculate the local image quality metrics (see Fig. 8).

**TABLE IV:** Phantom and Simulated Test Data for CUBDL Task 1

Data Info				Acquisition Info									Phantom Info	

Sequence #	Transmission type	# of angles	Plane wave angle range	US scanner	Transducer	Center Frequency [MHz]	Sampling Frequency [MHz]	Bandwith [MHz]	Aperture Width [mm]	Element Width [mm]	Pitch [mm]	Image Depth [mm]	Targets Included	Ref.

MYO001	Plane Wave	75	[−16, 16]	Verasonics Vantage 256	L11-4v	6.25	28.96	6.78	38.4	0.27	0.3	63.8	wire targets and inclusions	–
MYO002	Plane Wave	75	[−16, 16]	Verasonics Vantage 256	L11-4v	6.25	28.96	6.78	38.4	0.27	0.3	63.8	wire targets and inclusions	–
MYO003	Plane Wave	75	[−16, 16]	Verasonics Vantage 256	L11-4v	6.25	28.96	6.78	38.4	0.27	0.3	63.8	wire targets and inclusions	–
MYO004	Plane Wave	75	[−16, 16]	Verasonics Vantage 256	L11-4v	6.25	28.96	6.78	38.4	0.27	0.3	63.8	wire targets, inclusions, and clutter	–
MYO005	Plane Wave	75	[−16, 16]	Verasonics Vantage 256	L11-4v	6.25	28.96	6.78	38.4	0.27	0.3	63.8	wile targets, inclusions, and clutter	–

UFL001	Plane Wave	75	[−15, 15]	ULA-OP256	Esaote LA533	8	78.125	4.9	31.36	0.215	0.245	50	Hypoechoic target, grey scale target and wire targets	–
UFL002	Plane Wave	75	[−15, 15]	ULA-OP256	Esaote LA533	8	78.125	4.9	31.36	0.215	0.245	50	wire targets, grey scale targets	–
UFL004	Plane Wave	75	[−15, 15]	ULA-OP256	Esaote LA533	8	78.125	4.9	31.36	0.215	0.245	50	Wire targets	–
UFL005	Plane Wave	75	[−15, 15]	ULA-OP256	Esaote LA533	8	78.125	4.9	31.36	0.215	0.245	50	Hypoecoic targets	–

EUT003	Plane Wave	75	[−16, 16]	Verasonics Vantage 128	Philips S5-1	3.125	12.5	4	20.3	0.25	0.254	100	Wire targets	–
EUT006	Plane Wave	75	[−16, 16]	Verasonics Vantage 128	Philips S5-1	3.125	12.5	4	20.3	0.25	0.254	100	Wire targets	–

INS004	Plane Wave	75	[−16, 16]	Verasonics Vantage 256	L7-4	5.208	20.833	3.1248	37.846	0.25	0.298	56.7	Stiff inclusions	–
INS006	Plane Wave	75	[−16, 16]	Verasonics Vantage 256	L7-4	5.208	20.833	3.1248	37.846	0.25	0.298	56.7	Cysts	–
INS008	Plane Wave	75	[−16, 16]	Verasonics Vantage 256	L7-4	5.208	20.833	3.1248	37.846	0.25	0.298	56.7	Cysts and wire	–
INS015	Plane Wave	75	[−16, 16]	Verasonics Vantage 256	L12-5	7.813	31.25	4.6878	24.8	0.1703	0.1953	56.7	Vessel and wires with flow circulation	–
INS016	Plane Wave	75	[−16, 16]	Verasonics Vantage 256	L12-5	7.813	31.25	4.6878	24.8	0.1703	0.1953	56.7	Stiff inclusions	–
INS019	Plane Wave	75	[−16, 16]	Verasonics Vantage 256	L12-5	7.813	31.25	4.6878	24.8	0.1703	0.1953	56.7	Cysts	–
INS021	Plane Wave	75	[−16, 16]	Verasonics Vantage 256	L12-5	7.813	31.25	4.6878	24.8	0.1703	0.1953	56.7	Cysts + wire	–

OSL007	Plane Wave	75	[−16, 16]	Verasonics Vantage 256	L7-4	5.208333	20.866720	3.1248	38.100005	0.25	0.298	50	Cysts	–

OSL010	Plane Wave	75	[−16, 16]	Simulation Field II	N/A	5.13	20.8	–	38.1	–	0.3	50	Dynamic range phantom	[18]

**TABLE V:** *IN VIVO* Test Data for CUBDL Task 1

Data Info				Acquisition Info									*In Vivo* Info		
Sequence #	Transmission Type	Total # of plane waves	Angle Range	US scanner	Transducer	Center Frequency [MHz]	Sampling Frequency [MHz]	Bandwith [MHz]	Aperture Width [mm]	Element Width [mm]	Pitch [mm]	Image Depth [mm]	Data from	Organ	Ref.
TSH002	Plane Wave	31	[−15,15]	Verasonics Vantage 256	L10-5	7.5	6.25	5	38.37	0.27	0.3	40	Volunteer	Brachioradialis	[17]

**TABLE VI:** *IN VIVO* Data Acquired With Plane Wave Transmissions for the Post-CUBDL Analysis (Available With the Open Dataset)

Data Info				Acquisition Info									*In Vivo* Info		

Sequence #	Transmission Type	Total # of plane waves	Angle range	US scanner	Transducer	Center Frequency [MHz]	Sampling Frequency [MHz]	Bandwith [MHz]	Aperture Width [mm]	Element Width [mm]	Pitch [mm]	Image Depth [mm]	Data from	Organ	Ref.

JHU024	Plane Wave	73	[−8,8]	Alpinion ECUBE12-R	L8-17	12.5	40	9	25.6	0.11	0.2	30	Patient	Breast	[19]
JHU025	Plane Wave	73	[−8,8]	Alpinion ECUBE12-R	L8-17	12.5	40	9	25.6	0.11	0.2	30	Patient	Breast	[19]
JHU026	Plane Wave	73	[−8,8]	Alpinion ECUBE12-R	L8-17	12.5	40	9	25.6	0.11	0.2	30	Patient	Breast	[19]
JHU027	Plane Wave	73	[−8,8]	Alpinion ECUBE12-R	L8-17	12.5	40	9	25.6	0.11	0.2	30	Patient	Breast	[19]
JHU028	Plane Wave	75	[−16,16]	Alpinion ECUBE12-R	L8-17	12.5	40	9	25.6	0.11	0.2	30	Patient	Breast	[19]
JHU029	Plane Wave	75	[−16,16]	Alpinion ECUBE12-R	L8-17	12.5	40	9	25.6	0.11	0.2	30	Patient	Breast	[19]
JHU030	Plane Wave	73	[−8,8]	Alpinion ECUBE12-R	L8-17	12.5	40	9	25.6	0.11	0.2	30	Patient	Breast	[19]
JHU031	Plane Wave	75	[−16,16]	Alpinion ECUBE12-R	L8-17	12.5	40	9	25.6	0.11	0.2	30	Patient	Breast	[19]
JHU032	Plane Wave	75	[−16,16]	Alpinion ECUBE12-R	L8-17	12.5	40	9	25.6	0.11	0.2	30	Patient	Breast	[19]
JHU033	Plane Wave	75	[−16,16]	Alpinion ECUBE12-R	L3-8	5.5	40	5	38.4	0.24	0.3	30	Patient	Breast	[19]
JHU034	Plane Wave	75	[−16,16]	Alpinion ECUBE12-R	L3-8	5.5	40	5	38.4	0.24	0.3	30	Patient	Breast	[19]

**TABLE VII:** Remaining *IN VIVO* Data Acquired With Plane Wave Transmissions (Not Used for the CUBDL Task 1 Evaluations, But Available With the Open Dataset)

Data Info				Acquisition Info									*In Vivo* Info		
Sequence #	Transmission Type	Total # of plane waves	Angle range	US scanner	Transducer	Center Frequency [MHz]	Sampling Frequency [MHz]	Bandwith [MHz]	Aperture Width [mm]	Element Width [mm]	Pitch [mm]	Image Depth [mm]	Data from	Organ	Ref.
TSH003-TSH501	Plane Wave	31	[−15,15]	Verasinics Vantage 256	L10-5	7.5	6.25	5	38.37	0.27	0.3	40	Volunteer	Brachioradialis	[17]

**TABLE VIII:** Remaining Phantom Data Acquired With Plane Wave Transmissions (Not Used for the CUBDL Task 1 Evaluations, But Available With the Open Dataset)

Data Info				Acquisition Info									Phantom Info

Sequence #	Transmission type	# of angles	Plane wave angle range	US scanner	Transducer	Center Frequency [MHz]	Sampling Frequency [MHz]	Bandwith [MHz]	Aperture Width [mm]	Element Width [mm]	Pitch [mm]	Image Depth [mm]	Thrgets Included

MYO006	Plane Wave	75	[−16, 16]	Verasonics Vantage 256	L11-4v	6.25	28.96	6.78	38.4	0.27	0.3	63.8	Wire targets, inclusions, and clutter

UFL003	Plane Wave	75	[−15, 15]	ULA-OP256	Esaote LA533	8	78.125	4.9	31.36	0.215	0.245	50	Wire targets and hyperechoic target

EUT001	Plane Wave	75	[−16, 16]	Verasonics Vantage 128	Philips S5-1	3.125	12.5	4	20.3	0.25	0.254	100	Wire targets
EUT002	Plane Wave	75	[−16, 16]	Verasonics Vantage 128	Philips S5-1	3.125	12.5	4	20.3	0.25	0.254	100	Wire targets
EUT004	Plane Wave	75	[−16, 16]	Verasonics Vantage 128	Philips S5-1	3.125	12.5	4	20.3	0.25	0.254	100	Wire targets
EUT005	Plane Wave	75	[−16, 16]	Verasonics Vantage 128	Philips S5-1	3.125	12.5	4	20.3	0.25	0.254	100	Wire targets

INS001	Plane Wave	75	[−16, 16]	Verasonics Vantage 256	L7-4	5.208	20.833	3.1248	37.846	0.25	0.298	56.7	Vessel and wires
INS002	Plane Wave	75	[−16, 16]	Verasonics Vantage 256	L7-4	5.208	20.833	3.1248	37.846	0.25	0.298	56.7	Vessel and wires with flow circulation
INS003	Plane Wave	75	[−16, 16]	Verasonics Vantage 256	L7-4	5.208	20.833	3.1248	37.846	0.25	0.298	56.7	Homogeneous
INS005	Plane Wave	75	[−16, 16]	Verasonics Vantage 256	L7-4	5.208	20.833	3.1248	37.846	0.25	0.298	56.7	Wires
INTS007	Plane Wave	75	[−16, 16]	Verasonics Vantage 256	L7-4	5.208	20.833	3.1248	37.846	0.25	0.298	56.7	Wires
INS009	Plane Wave	75	[−16, 16]	Verasonics Vantage 256	L7-4	5.208	20.833	3.1248	37.846	0.25	0.298	56.7	Inclusions
INS010	Plane Wave	75	[−16, 16]	Verasonics Vantage 256	L7-4	5.208	20.833	3.1248	37.846	0.25	0.298	56.7	Inclusions
INS011	Plane Wave	75	[−16, 16]	Verasonics Vantage 256	L7-4	5.208	20.833	3.1248	37.846	0.25	0.298	56.7	Brain structure
INS012	Plane Wave	75	[−16, 16]	Verasonics Vantage 256	L7-4	5.208	20.833	3.1248	37.846	0.25	0.298	56.7	Brain structure
INS013	Plane Wave	75	[−16, 16]	Verasonics Vantage 256	L7-4	5.208	20.833	3.1248	37.846	0.25	0.298	56.7	Vessel
INS014	Plane Wave	75	[−16, 16]	Verasonics Vantage 256	L12-5	7.813	31.25	4.6878	24.8	0.1703	0.1953	56.7	Vessel and wires
INS017	Plane Wave	75	[−16, 16]	Verasonics Vantage 256	L12-5	7.813	31.25	4.6878	24.8	0.1703	0.1953	56.7	Homogeneous
INS018	Plane Wave	75	[−16, 16]	Verasonics Vantage 256	L12-5	7.813	31.25	4.6878	24.8	0.1703	0.1953	56.7	Wires
INS020	Plane Wave	75	[−16, 16]	Verasonics Vantage 256	L12-5	7.813	31.25	4.6878	24.8	0.1703	0.1953	56.7	Wires
INS022	Plane Wave	75	[−16, 16]	Verasonics Vantage 256	L12-5	7.813	31.25	4.6878	24.8	0.1703	0.1953	56.7	Inclusions
INS023	Plane Wave	75	[−16, 16]	Verasonics Vantage 256	L12-5	7.813	31.25	4.6878	24.8	0.1703	0.1953	56.7	Inclusions
INS024	Plane Wave	75	[−16, 16]	Verasonics Vantage 256	L12-5	7.813	31.25	4.6878	24.8	0.1703	0.1953	56.7	Brain structure
INS025	Plane Wave	75	[−16, 16]	Verasonics Vantage 256	L12-5	7.813	31.25	4.6878	24.8	0.1703	0.1953	56.7	Brain structure
INS026	Plane Wave	75	[−16, 16]	Verasonics Vantage 256	L12-5	7.813	31.25	4.6878	24.8	0.1703	0.1953	56.7	Vessel

OSL002	Plane Wave	75	[−16, 16]	Verasonics Vantage 256	L7-4	5.208333	20.866720	3.1248	38.100000	0.25	0.298	50	Wires
OSL003	Plane Wave	75	[−16, 16]	Verasonics Vantage 256	L7-4	5.208333	20.866720	3.1248	38.100001	0.25	0.298	50	Wires and cysts
OSL004	Plane Wave	75	[−16, 16]	Verasonics Vantage 256	L7-4	5.208333	20.866720	3.1248	38.100002	0.25	0.298	50	Wires and cysts
OSL005	Plane Wave	75	[−16, 16]	Verasonics Vantage 256	L7-4	5.208333	20.866720	3.1248	38.100003	0.25	0.298	50	Cysts
OSL006	Plane Wave	75	[−16, 16]	Verasonics Vantage 256	L7-4	5.208333	20.866720	3.1248	38.100004	0.25	0.298	50	Cysts

**TABLE IX:** Phantom Data and Simulated Data Acquired With Focused Transmissions (Crowd-Sourced for CUBDL Task 3 and Available With the Open Dataset)

Data Info					Acquisition Info									Phantom Info	

Sequence #	Transmission type	# of steering angles	Steering angle range	Focal Depth [mm]	US scanner	Transducer	Center Frequency [MHz]	Sampling Frequency [MHz]	Bandwith [MHz]	Aperture Width [mm]	Element Width [mm]	Pitch [mm]	Image Depth [mm]	Targets Included	Ref.

OSL001	Focused	–	–	22	Simulation Field II	N/A	5.13	100	–	38.1	–	0.3	50	Dynamic range phantom	[54]

OSL015	Focused	–	–	50	Verasonics Vantage 256	L11-5	5.00	20.42	3.12	38.10	0.25	0.298	50	Cyst and point targets	[54]

JHU019	Focused	128	[−45,45]	50	Verasonics Vantage 128	P4-2v	2.97	11.90	3	18.90	0.295	0.3	50	Point targets	–
JHU020	Focused	128	[−45,45]	60	Verasonics Vantage 128	P4-2v	2.97	11.90	3	18.90	0.295	0.3	60	Cyst and point targets	–
JHU021	Focused	128	[−45,45]	85	Verasonics Vantage 128	P4-2v	2.97	11.90	3	18.90	0.295	0.3	85	Cyst and point targets	–
JHU022	Focused	128	[−45,45]	73	Verasonics Vantage 128	P4-2v	2.97	11.90	3	18.90	0.295	0.3	73	Cyst	–
JHU023	Focused	128	[−45,45]	36	Verasonics Vantage 128	P4-2v	2.97	11.904762	3	18.90	0.295	0.3	36	Cyst and point targets	–

**TABLE X:** *IN VIVO* Data Acquired With Focused Transmissions (Crowd-Sourced for CUBDL Task 3 and Available With the Open Dataset)

Data Info					Acquisition Info									*In Vivo* Info		

Sequence #	Transmission type	# of steering angles	Steering angle range	Focal Depth [mm]	US scanner	Transducer	Center Frequency [MHz]	Sampling Frequency [MHz]	Bandwith [MHz]	Aperture Width [mm]	Element Width [mm]	Pitch [mm]	Image Depth [mm]	Data from	Organ	Ref.

JHU001	Focused	–	–	20	Alpinion ECUBE12-R	L3-8	5.5	40	5	38.1	0.24	0.3	40	Patient	Breast	[30]
JHU002	Focused	–	–	20	Alpinion ECUBE12-R	L8-17	12.5	40	9	25.4	0.11	0.2	40	Patient	Breast	[30]

OSL008	Focused	–	–	22	Verasonics Vantage 256	L7-4	5.2	20.8	3.1248	37.8	0.25	0.298	40	Volunteer	Carotid	[53]
OSL009	Focused	–	–	22	Verasonics Vantage 256	L7-4	5.2	20.8	3.1248	37.8	0.25	0.298	40	Volunteer	Carotid	[53]
OSL011	Focused	101	[−37.5, 37.5]	67	Verasonics Vantage 256	P4-2	3.0	11.9	3	18.9	0.295	0.3	120	Volunteer	Heart	[31]
OSL012	Focused	101	[−37.5, 37.5]	52	Verasonics Vantage 256	P7-4	3.0	11.9	3	18.9	0.295	0.3	120	Volunteer	Heart	[31]
OSL013	Focused	101	[−37.5, 37.5]	52	Verasonics Vantage 256	P4-2	3.0	11.9	3	18.9	0.295	0.3	120	Volunteer	Heart	[31]
OSL014	Focused	101	[−37.5, 37.5]	52	Verasonics Vantage 256	P4-2	3.0	11.9	3	18.9	0.295	0.3	120	Volunteer	Heart	[31]

## References

[1] Liu S , “Deep learning in medical ultrasound analysis: A review,” Engineering, vol. 5, no. 2, pp. 261–275, Apr. 2019.

[2] van Sloun RJ, Cohen R, and Eldar YC, “Deep learning in ultrasound imaging,” Proc. IEEE, vol. 108, no. 1, pp. 11–29, Jan. 2020.

[3] Shan C, Tan T, Wu S, and Schnabel JA, “Guest editorial: Deep learning in ultrasound imaging,” IEEE J. Biomed. Health Informat, vol. 24, no. 4, pp. 929–930, Apr. 2020.

[4] Mischi M, Bell MAL, van Sloun RJ, and Eldar YC, “Deep learning in medical ultrasound-from image formation to image analysis,” IEEE Trans. Ultrason., Ferroelectr., Freq. Control, vol. 67, no. 12, pp. 2477–2480, 2020.

[5] Nair AA, Tran TD, Reiter A, and Bell MAL, “A deep learning based alternative to beamforming ultrasound images,” in Proc. IEEE Int. Conf. Acoust., Speech Signal Process (ICASSP), Apr. 2018, pp. 3359–3363.

[6] Bhatt M, Nair AA, Kempski KM, and Lediju Bell MA, “Multi-task learning for ultrasound image formation and segmentation directly from raw *in vivo* data,” in Proc. IEEE Int. Ultrason. Symp (IUS), Sep. 2020, pp. 1–4.

[7] Tierney J, Luchies A, Berger M, and Byram B, “Evaluating input domain and model selection for deep network ultrasound beamforming,” IEEE Trans. Ultrason., Ferroelectr., Freq. Control, vol. 68, no. 7, pp. 2370–2385, Jul. 2021. 33684036 10.1109/TUFFC.2021.3064303 PMC8285087

[8] Wiacek A, Gonzalez E, and Bell MAL, “CohereNet: A deep learning architecture for ultrasound spatial correlation estimation and coherence-based beamforming,” IEEE Trans. Ultrason., Ferroelectr., Freq. Control, vol. 67, no. 12, pp. 2574–2583, Dec. 2020. 32203018 10.1109/TUFFC.2020.2982848 PMC8034551

[9] Luijten B , “Adaptive ultrasound beamforming using deep learning,” IEEE Trans. Med. Imag, vol. 39, no. 12, pp. 3967–3978, Dec. 2020. 10.1109/TMI.2020.3008537 32746139

[10] Khan S, Huh J, and Ye JC, “Adaptive and compressive beamforming using deep learning for medical ultrasound,” IEEE Trans. Ultrason., Ferroelectr., Freq. Control, vol. 67, no. 8, pp. 1558–1572, Aug. 2020. 32149628 10.1109/TUFFC.2020.2977202

[11] Hyun D, Brickson LL, Looby KT, and Dahl JJ, “Beamforming and speckle reduction using neural networks,” IEEE Trans. Ultrason., Ferroelectr., Freq. Control, vol. 66, no. 5, pp. 898–910, May 2019. 30869612 10.1109/TUFFC.2019.2903795 PMC7012504

[12] Russakovsky O , “ImageNet large scale visual recognition challenge,” Int. J. Comput. Vis, vol. 115, no. 3, pp. 211–252, Dec. 2015.

[13] Murphy K , “Evaluation of registration methods on thoracic CT: The EMPIRE10 challenge,” IEEE Trans. Med. Imag, vol. 30, no. 11, pp. 1901–1920, Nov. 2011. 10.1109/TMI.2011.2158349 21632295

[14] Accessed: Jul. 1, 2021. [Online]. Available: https://cubdl.jhu.edu/

[15] Accessed: Jul. 1, 2021. [Online]. Available: https://gitlab.com/dongwoon.hyun/cubdl

[16] Bell MAL , “Challenge on ultrasound beamforming with deep learning (CUBDL) datasets,” doi: 10.21227/f0hn-8f92.

[17] Zhang X, Li J, He Q, Zhang H, and Luo J, “High-quality reconstruction of plane-wave imaging using generative adversarial network,” in Proc. IEEE Int. Ultrason. Symp (IUS), Oct. 2018, pp. 1–4.

[18] PICMUS. (2020). Plane-Wave Imaging Evaluation Framework for Medical Ultrasound. [Online]. Available: https://www.creatis.insa-lyon.fr/EvaluationPlatform/picmus/

[19] Li Z, Wiacek A, and Bell MAL, “Beamforming with deep learning from single plane wave RF data,” in Proc. IEEE Int. Ultrason. Symp (IUS), Sep. 2020, pp. 1–4.

[20] Bell MAL, Huang J, Hyun D, Eldar YC, van Sloun R, and Mischi M, “Challenge on ultrasound beamforming with deep learning (CUBDL),” in Proc. IEEE Int. Ultrason. Symp (IUS), Sep. 2020, pp. 1–5, doi: 10.1109/IUS46767.2020.9251434.

[21] Liebgott H, Rodriguez-Molares A, Cervenansky F, Jensen JA, and Bernard O, “Plane-wave imaging challenge in medical ultrasound,” in Proc. IEEE Int. Ultrason. Symp (IUS), Sep. 2016, pp. 1–4.

[22] Dong E, Du H, and Gardner L, “An interactive Web-based dashboard to track COVID-19 in real time,” Lancet Infectious Diseases, vol. 20, no. 5, pp. 533–534, May 2020. 32087114 10.1016/S1473-3099(20)30120-1 PMC7159018

[23] Nair AA, Washington KN, Tran TD, Reiter A, and Bell MAL, “Deep learning to obtain simultaneous image and segmentation outputs from a single input of raw ultrasound channel data,” IEEE Trans. Ultrason., Ferroelectr., Freq. Control, vol. 67, no. 12, pp. 2493–2509, Dec. 2020. 32396084 10.1109/TUFFC.2020.2993779 PMC7990652

[24] Howard AG , “MobileNets: Efficient convolutional neural networks for mobile vision applications,” 2017, arXiv:1704.04861. [Online]. Available: http://arxiv.org/abs/1704.04861

[25] Iandola FN, Han S, Moskewicz MW, Ashraf K, Dally WJ, and Keutzer K, “SqueezeNet: AlexNet-level accuracy with 50x fewer parameters and <0.5MB model size,” 2016, arXiv:1602.07360. [Online]. Available: http://arxiv.org/abs/1602.07360

[26] Kawatsu C , “Gesture recognition for robotic control using deep learning,” in Proc. NDIA Ground Vehicle Syst. Eng. Technol. Symp, 2017, pp. 1–7.

[27] Wang Y, Kempski K, Kang JU, and Bell MAL, “A conditional adversarial network for single plane wave beamforming,” in Proc. IEEE Int. Ultrason. Symp (IUS), Sep. 2020, pp. 1–4.

[28] Rothlubbers S , “Improving image quality of single plane wave ultrasound via deep learning based channel compounding,” in Proc. IEEE Int. Ultrason. Symp (IUS), Sep. 2020, pp. 1–4.

[29] Goudarzi S, Asif A, and Rivaz H, “Ultrasound beamforming using MobileNetV2,” in Proc. IEEE Int. Ultrason. Symp (IUS), Sep. 2020, pp. 1–4.

[30] Wiacek A , “Robust short-lag spatial coherence imaging of breast ultrasound data: Initial clinical results,” IEEE Trans. Ultrason., Ferroelectr., Freq. Control, vol. 66, no. 3, pp. 527–540, Mar. 2019. 30507500 10.1109/TUFFC.2018.2883427 PMC7730490

[31] Rindal OMH, Aakhus S, Holm S, and Austeng A, “Hypothesis of improved visualization of microstructures in the interventricular septum with ultrasound and adaptive beamforming,” Ultrasound Med. Biol, vol. 43, no. 10, pp. 2494–2499, Oct. 2017. 28689675 10.1016/j.ultrasmedbio.2017.05.023

[32] Duck FA, “Acoustic properties of tissue at ultrasonic frequencies,” in Physical Properties of Tissue: A Comprehensive Reference Book. San Diego, CA, USA: Academic, 1990, pp. 73–124.

[33] Park B, Whittaker AD, Miller RK, and Hale DS, “Predicting intramuscular fat in beef longissimus muscle from speed of sound,” J. Animal Sci, vol. 72, no. 1, pp. 109–116, Jan. 1994. 10.2527/1994.721109x 8138477

[34] Ghoshal G, Luchies AC, Blue JP, and Oelze ML, “Temperature dependent ultrasonic characterization of biological media,” J. Acoust. Soc. Amer, vol. 130, no. 4, pp. 2203–2211, Oct. 2011. 21973375 10.1121/1.3626162 PMC3206913

[35] Nock L, Trahey GE, and Smith SW, “Phase aberration correction in medical ultrasound using speckle brightness as a quality factor,” J. Acoust. Soc. Amer, vol. 85, no. 5, pp. 1819–1833, May 1989. 2732378 10.1121/1.397889

[36] Anderson ME, McKeag MS, and Trahey GE, “The impact of sound speed errors on medical ultrasound imaging,” J. Acoust. Soc. Amer, vol. 107, no. 6, pp. 3540–3548, Jun. 2000. 10875398 10.1121/1.429422

[37] Mallart R and Fink M, “Adaptive focusing in scattering media through sound-speed inhomogeneities: The van Cittert Zernike approach and focusing criterion,” J. Acoust. Soc. Amer, vol. 96, no. 6, pp. 3721–3732, Dec. 1994.

[38] Flax SW and O’Donnell M, “Phase-aberration correction using signals from point reflectors and diffuse scatterers: Basic principles,” IEEE Trans. Ultrason., Ferroelectr., Freq. Control, vol. 35, no. 6, pp. 758–767, Nov. 1988. 18290213 10.1109/58.9333

[39] Anderson ME and Trahey GE, “The direct estimation of sound speed using pulse-echo ultrasound,” J. Acoust. Soc. Amer, vol. 104, no. 5, pp. 3099–3106, Nov. 1998. 9821351 10.1121/1.423889

[40] Imbault M , “Robust sound speed estimation for ultrasound-based hepatic steatosis assessment,” Phys. Med. Biol, vol. 62, no. 9, p. 3582, 2017. 28225357 10.1088/1361-6560/aa6226

[41] Perrot V, Polichetti M, Varray F, and Garcia D, “So you think you can DAS? A viewpoint on delay-and-sum beamforming,” Ultrasonics, vol. 111, Mar. 2021, Art. no. 106309. 33360053 10.1016/j.ultras.2020.106309

[42] Paszke A , “Pytorch: An imperative style, high-performance deep learning library,” in Proc. Adv. Neural Inf. Process. Syst, Wallach H, Larochelle H, Beygelzimer A, Alché-Buc F. d’, Fox E, and Garnett R, Eds. Red Hook, NY, USA: Curran Associates, 2019, pp. 8024–8035. [Online]. Available: http://papers.neurips.cc/paper/9015-pytorch-an-imperative-style-high-pe%rformance-deep-learning-library.pdf

[43] Jensen JA, “FIELD: A program for simulating ultrasound systems,” in Proc. IEEE 10th Nordic-Baltic Conf. Biomed. Imag, vol. 34, Mar. 1996, pp. 351–353.

[44] Jensen JA and Svendsen NB, “Calculation of pressure fields from arbitrarily shaped, apodized, and excited ultrasound transducers,” IEEE Trans. Ultrason., Ferroelectr., Freq. Control, vol. 39, no. 2, pp. 262–267, Mar. 1992. 18263145 10.1109/58.139123

[45] Yang C, Jiao Y, Jiang T, Xu Y, and Cui Y, “A united sign coherence factor beamformer for coherent plane-wave compounding with improved contrast,” Appl. Sci, vol. 10, no. 7, p. 2250, Mar. 2020.

[46] Wang Z, Simoncelli EP, and Bovik AC, “Multiscale structural similarity for image quality assessment,” in Proc. 37th Asilomar Conf. Signals, Syst. Comput, Nov. 2003, pp. 1398–1402.

[47] Sandler M, Howard A, Zhu M, Zhmoginov A, and Chen L-C, “MobileNetV2: Inverted residuals and linear bottlenecks,” in Proc. IEEE/CVF Conf. Comput. Vis. Pattern Recognit, Jun. 2018, pp. 4510–4520.

[48] Synnevag J-F, Austeng A, and Holm S, “Benefits of minimum-variance beamforming in medical ultrasound imaging,” IEEE Trans. Ultrason., Ferroelectr., Freq. Control, vol. 56, no. 9, pp. 1868–1879, Sep. 2009. 19811990 10.1109/TUFFC.2009.1263

[49] Loshchilov I and Hutter F, “Decoupled weight decay regularization,” 2017, arXiv:1711.05101. [Online]. Available: http://arxiv.org/abs/1711.05101

[50] Rodriguez-Molares A , “The ultrasound toolbox,” in Proc. IEEE Int. Ultrason. Symp (IUS), Sep. 2017, pp. 1–4.

[51] Rodriguez-Molares A , “The generalized Contrast-to-Noise ratio: A formal definition for lesion detectability,” IEEE Trans. Ultrason., Ferroelectr., Freq. Control, vol. 67, no. 4, pp. 745–759, Apr. 2020. 31796398 10.1109/TUFFC.2019.2956855 PMC8354776

[52] Rindal OMH, Austeng A, Fatemi A, and Rodriguez-Molares A, “The effect of dynamic range alterations in the estimation of contrast,” IEEE Trans. Ultrason., Ferroelectr., Freq. Control, vol. 66, no. 7, pp. 1198–1208, Jul. 2019. 30990429 10.1109/TUFFC.2019.2911267

[53] Rodriguez-Molares A, Rindal OMH, D’hooge J, Masoy S-E, Austeng A, and Torp H, “The generalized Contrast-to-Noise ratio,” in Proc. IEEE Int. Ultrason. Symp (IUS), Oct. 2018, pp. 1–4. 10.1109/TUFFC.2019.2956855 PMC8354776 31796398

[54] Lediju MA, Pihl MJ, Dahl JJ, and Trahey GE, “Quantitative assessment of the magnitude, impact and spatial extent of ultrasonic clutter,” Ultrason. Imag, vol. 30, no. 3, pp. 151–168, Jul. 2008. 10.1177/016173460803000302 PMC3306837 19149461

[55] Vedula S, Senouf O, Zurakhov G, Bronstein A, Michailovich O, and Zibulevsky M, “Learning beamforming in ultrasound imaging,” in Proc. 2nd Int. Conf. Med. Imag. With Deep Learn, vol. 102, Cardoso MJ, Feragen A, Glocker B, Konukoglu E, Oguz I, Unal G, and Vercauteren T, Eds. New York, NY, USA: PMLR, Jul. 2019, pp. 493–511. [Online]. Available: http://proceedings.mlr.press/v102/vedula19a.html

[56] Jarosik P, Byra M, and Lewandowski M, “WaveFlow-towards integration of ultrasound processing with deep learning,” in Proc. IEEE Int. Ultrason. Symp (IUS), Oct. 2018, pp. 1–3.

[57] Hyun D, Li YL, Steinberg I, Jakovljevic M, Klap T, and Dahl JJ, “An open source GPU-based beamformer for real-time ultrasound imaging and applications,” in Proc. IEEE Int. Ultrason. Symp (IUS), Oct. 2019, pp. 20–23.

[58] Zhang J, He Q, Xiao Y, Zheng H, Wang C, and Luo J, “Ultrasound image reconstruction from plane wave radio-frequency data by self-supervised deep neural network,” Med. Image Anal, vol. 70, May 2021, Art. no. 102018. 33711740 10.1016/j.media.2021.102018

[59] Zhou Z, Guo Y, and Wang Y, “Ultrasound deep beamforming using a multiconstrained hybrid generative adversarial network,” Med. Image Anal, vol. 71, Jul. 2021, Art. no. 102086. 33979760 10.1016/j.media.2021.102086

[60] Qi Y, Guo Y, and Wang Y, “Image quality enhancement using a deep neural network for plane wave medical ultrasound imaging,” IEEE Trans. Ultrason., Ferroelectr., Freq. Control, vol. 68, no. 4, pp. 926–934, Apr. 2021. 32915734 10.1109/TUFFC.2020.3023154

[61] P Mathews R and Panicker MR, “Towards fast region adaptive ultrasound beamformer for plane wave imaging using convolutional neural networks,” 2021, arXiv:2106.07006. [Online]. Available: http://arxiv.org/abs/2106.07006 10.1109/EMBC46164.2021.9630930 34891854

[62] Tang J, Zou B, Li C, Feng S, and Peng H, “Plane-wave image reconstruction via generative adversarial network and attention mechanism,” IEEE Trans. Instrum. Meas, vol. 70, pp. 1–15, 2021.33776080

[63] Maier-Hein L , “Why rankings of biomedical image analysis competitions should be interpreted with care,” Nature Commun, vol. 9, no. 1, pp. 1–13, 2018. 30523263 10.1038/s41467-018-07619-7 PMC6284017

